# High Prevalence and Clinical Relevance of Genes Affected by Chromosomal Breaks in Colorectal Cancer

**DOI:** 10.1371/journal.pone.0138141

**Published:** 2015-09-16

**Authors:** Evert van den Broek, Maurits J. J. Dijkstra, Oscar Krijgsman, Daoud Sie, Josien C. Haan, Joleen J. H. Traets, Mark A. van de Wiel, Iris D. Nagtegaal, Cornelis J. A. Punt, Beatriz Carvalho, Bauke Ylstra, Sanne Abeln, Gerrit A. Meijer, Remond J. A. Fijneman

**Affiliations:** 1 Department of Pathology, VU University Medical Center, Amsterdam, The Netherlands; 2 Department of Computer Science, VU University, Amsterdam, The Netherlands; 3 Department of Epidemiology & Biostatistics, VU University Medical Center, Amsterdam, The Netherlands; 4 Department of Mathematics, VU University, Amsterdam, The Netherlands; 5 Department of Pathology, Radboud University Nijmegen Medical Center, Nijmegen, The Netherlands; 6 Department of Medical Oncology, Academic Medical Center, Amsterdam, The Netherlands; National Cancer Center, JAPAN

## Abstract

**Background:**

Cancer is caused by somatic DNA alterations such as gene point mutations, DNA copy number aberrations (CNA) and structural variants (SVs). Genome-wide analyses of SVs in large sample series with well-documented clinical information are still scarce. Consequently, the impact of SVs on carcinogenesis and patient outcome remains poorly understood. This study aimed to perform a systematic analysis of genes that are affected by CNA-associated chromosomal breaks in colorectal cancer (CRC) and to determine the clinical relevance of recurrent breakpoint genes.

**Methods:**

Primary CRC samples of patients with metastatic disease from CAIRO and CAIRO2 clinical trials were previously characterized by array-comparative genomic hybridization. These data were now used to determine the prevalence of CNA-associated chromosomal breaks within genes across 352 CRC samples. In addition, mutation status of the commonly affected *APC*, *TP53*, *KRAS*, *PIK3CA*, *FBXW7*, *SMAD4*, *BRAF* and *NRAS* genes was determined for 204 CRC samples by targeted massive parallel sequencing. Clinical relevance was assessed upon stratification of patients based on gene mutations and gene breakpoints that were observed in >3% of CRC cases.

**Results:**

In total, 748 genes were identified that were recurrently affected by chromosomal breaks (FDR <0.1). *MACROD2* was affected in 41% of CRC samples and another 169 genes showed breakpoints in >3% of cases, indicating that prevalence of gene breakpoints is comparable to the prevalence of well-known gene point mutations. Patient stratification based on gene breakpoints and point mutations revealed one CRC subtype with very poor prognosis.

**Conclusions:**

We conclude that CNA-associated chromosomal breaks within genes represent a highly prevalent and clinically relevant subset of SVs in CRC.

## Introduction

Cancer is caused by genomic aberrations that drive tumor initiation and progression. Oncogene activation and tumor suppressor gene inactivation can be caused by several classes of somatic DNA aberrations, including non-synonymous (point) mutations, chromosomal copy number aberrations (CNAs) and structural variants (SVs) [1]. SVs represent deletions, insertions, inversions, and intra- and inter-chromosomal translocations, all of which involve chromosomal breaks [2]. Interestingly, while point mutations and DNA copy number changes have been examined extensively, the effects of genes with chromosomal breaks are poorly characterized. Taking colorectal cancer (CRC) as an example, to date several in-frame fusion genes have been reported including *VTI1A-TCF7L2*, *NAV2-TCF7L1* and the R-spondin fusions *PTPRK-RSPO3* and *EIF3E-RSPO2* [3–5]. The R-spondin fusions activate the Wnt signaling pathway and are mutually exclusive with *APC* mutations, indicating that these translocations cause gain-of-function protein alterations. Alternatively, SVs can also cause loss-of-function alterations. For example, deletion of the stop codon of the *EPCAM* gene results in a transcriptional read-through that causes hypermethylation and consequently silencing of the adjacent mismatch repair gene *MSH2* [6]. However, despite these intriguing examples, thorough investigation of SVs in CRC has been hampered by the lack of whole genome deep sequencing information on large series of samples. Consequently, the putative impact of SVs in (colorectal) tumor development is probably highly underestimated.

We previously performed high-resolution array-comparative genomic hybridization (array-CGH) analysis on a series of approximately 350 primary CRC samples from patients who had developed metastatic disease and participated in the CAIRO and CAIRO2 phase III clinical trials [7–9]. In the present study we used these data to determine the genomic positions of chromosomal breakpoints, based on the assumption that intra-chromosomal changes in CNA-status can only be explained by mechanisms that involve chromosomal breaks. Although such an analysis does not provide a comprehensive overview of SVs in the cancer genome, we hypothesized that this analysis would identify a substantial subset of SVs at a sufficient resolution to allocate chromosomal breaks to gene positions. Moreover, we anticipated that non-random recurrent events among CRC samples would reveal genes that enhance tumor development. Here, we show that this approach revealed 748 recurrent breakpoint genes and demonstrate their impact on CRC classification.

## Materials and Methods

### Copy number aberration-associated chromosomal breakpoint detection

**Fig 1:**
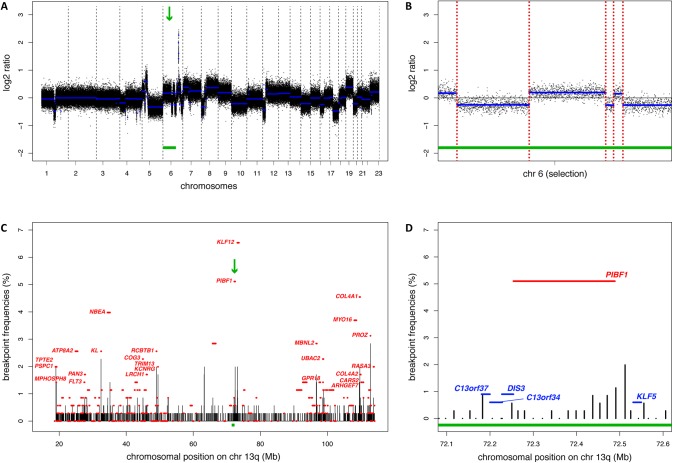
CNA-associated gene breakpoint detection. (A) Array-CGH DNA copy number profile of one CRC sample. The X-axis depicts chromosomes 1–22 and X (numbered 23) with chromosome boundaries indicated by vertical dotted lines. The Y-axis depicts the log2 ratio of the amount of tumor DNA *versus* patient-matched normal DNA. Black dots represent individual array-CGH probe datapoints. The blue horizontal lines represent DNA segments, indicative for tumor DNA copy number aberrations when deviating from 0. The green arrow and bar indicate the region of chromosome 6 that is highlighted in Fig 1B. (B) Enlargement of Fig 1A (green bar). Vertical dotted red lines indicate genomic locations of CNA-associated chromosomal breakpoints, *i*.*e*. the genomic positions where log2 ratios of DNA segments change. (C) Frequency plot of CNA-associated chromosomal breakpoints on the q-arm of chromosome 13. The X-axis depicts the genomic position in Mb. The Y-axis depicts the chromosomal breakpoint frequencies across the cohort of 352 CRC samples. Breakpoint frequencies are indicated on array-CGH probe-level (vertical black bars) and on gene-level (horizontal red bars). Recurrent breakpoint genes (FDR<0.1) are named. The green arrow and bar indicate the *PIBF1* region of chromosome 13q that is highlighted in Fig 1D. (D) Enlargement of Fig 1C (green bar), which illustrates that *PIBF1* gene breakpoints are concentrated at the distal part of the gene. Neighbouring genes that do not harbor significant breakpoint recurrence rates are indicated in blue.

Patients selected for the current study participated in either of the two multicentre phase III trials of the Dutch Colorectal Cancer Group (DCCG), namely CAIRO (CKTO 2002–07, ClinicalTrials.gov; NCT00312000) and CAIRO2 (CKTO 2005–02, ClinicalTrials.gov; NCT00208546). The two randomized clinical trials were approved by the Committee on Human-Related Research Arnhem—Nijmegen and by the local institutional review boards. The written informed consent required for all patients before study entry also included translational research on tumour tissue. CNA-associated chromosomal breakpoint detection was performed across 352 CRC samples. Array-CGH data were previously obtained using DNA isolated from formalin-fixed paraffin-embedded (FFPE) primary tumors and patient-matched normal tissue pairs [9]. The 4548 probes that had been added to enrich for the coverage of 238 Cancer Census genes were now excluded for chromosomal breakpoint analysis, leaving 168823 probes that were evenly distributed across the genome at approximately 17kb intervals (S1 Table). Genomic probe positions were based on human genome NCBI Build36/hg18. Array-CGH data have been deposited in NCBI’s Gene Expression Omnibus (GEO accession number GSE63216). DNA copy number segments were defined by the R-package “DNAcopy” (version 1.36.0) and were demarcated by the first and last probe of the segment [10]. Chromosomal breakpoints were defined by the genomic start positions of DNA copy number segments (Fig 1B) with the exception of the first DNA segment of each chromosome and of breakpoints between two copy number neutral regions as defined by the R-package “CGHcall” (version 2.17.6) [11]. To detect genes that were affected by CNA-associated chromosomal breaks, breakpoints were mapped to gene annotations based on human reference genome hg18/Ensembl54.

### Statistical analysis of chromosomal breakpoint detection

A dedicated statistical significance analysis was devised for the gene-based chromosomal breakpoint analysis, consisting of three steps. First, per array-CGH profile the baseline probability of a breakpoint occurring in a gene at random was determined, accounting for the number of breakpoints in a profile, gene length by gene-associated probe coverage and the number of gene-associated probes using a logistic regression. Second, the test statistic was defined as the number of profiles with at least one breakpoint in a given gene. Then, a *P*-value was computed from the null-distribution of the test statistic. This null-distribution was a convolution (over independent profiles) of Bernoulli random variables with a gene- and profile-specific ‘success (= breakpoint) probability’. Third, to all *P*-values of the candidate breakpoint genes, multiple testing was applied by a dedicated Benjamini-Hochberg-type FDR correction [12]. This correction accounts for the discreteness of the null-distribution. The probe-based chromosomal breakpoint statistical analysis was performed under the assumption that per array-CGH profile the probability to be a CNA-associated breakpoint probe is equal across probes. In this case the dedicated Benjamini-Hochberg-type FDR correction is equivalent to the standard Benjamini-Hochberg FDR correction, because, unlike for the genes, all probes correspond to the same null-distribution. FDR less than 0.1 was considered significant.

### Gene mutation analysis

*APC*, *TP53*, *KRAS*, *PIK3CA*, *FBXW7*, *SMAD4*, *BRAF* and *NRAS* are genes with a published mutation prevalence in CRC of approximately 3% or more [4]. FFPE DNA samples were analyzed by next generation sequencing using the TruSeq Amplicon Cancer Panel (TSACP; Illumina Inc, San Diego, CA USA). Gene mutation status was determined using the variant caller pipeline “Falco” [13]. Reads were aligned to the human reference genome (NCBI Build37/hg19) and variants were annotated to dbSNP entries (build 137). Mutations were called when the annotated variant was observed in at least 20% of the reads and was designated as a non-synonymous aberration.

### Network Based Stratification

NBS was used to cluster CRC samples, while including information from gene breakpoint and gene mutation molecular interactions [14]. The baseline clinicopathological characteristics of the CRC samples (n = 203, see S5 Table) were similar to the series analyzed by Haan et al. [9]. The *SMAD4* gene acquired both breakpoints and mutations, which were merged for NBS analysis. For the network propagation step the predefined STRING human protein interaction network was used as supplied with the NBS distribution. NBS parameters were set to their default values except for *k* that was set to 4. Using the sample-similarity matrix from NBS, samples were assigned to CRC subtypes by average linkage hierarchical clustering. CRC patients were clustered into four CRC subtypes and OS rates were visualized by Kaplan-Meier curves and corresponding *P*-values were calculated by log-rank testing.

### CRC subtype-associated genes

NBS does not provide network-based gene aberration scores as standard output. Therefore, to determine what genes were significantly associated with a specific CRC subtype, the network-based gene aberration scores for each gene per sample were extracted as follows. First, for every NBS iteration *i* of in total *n* iterations (n = 1000) the input matrices *V*_*i*_ were reconstructed from the factor matrices *W*_*i*_ and *H*_*i*_ that were obtained during the non-negative matrix factorization procedure:

$$V_{i}=\;W_{i}H_{i}$$

The matrices, *V*_*i*_, represent the data used by NBS to determine the sample clustering for every iteration. An averaged vector of network-based gene aberration scores, *R*_*s*_ was now obtained for every sample *s* by averaging over the input matrices *V*_*i*_ across all *n* iterations:

$$R_{s}=\frac{1}{C_{s}}\left[\sum_{i=1}^{n}V_{is}\right]$$

Here, *V*_*is*_ is the row from *V*_*s*_ that corresponds to sample *s* in iteration *i*. *C*_*s*_ is a normalization factor, defined as the number of iterations in which a sample *s* was selected for clustering. Note that if a sample was not selected during clustering all *V*_*is*_ values are set to zero. Mann-Whitney U tests were performed over the averaged network-based gene-aberration scores to test if a specific gene contributed to the formation of a CRC subtype. For every gene these *R*_*s*_ scores were grouped according to the CRC subtype to determine the *P*-values, indicating whether a gene contributed significantly to the formation of a specific CRC subtype.

### Multi-Dendrix

Data input for Multi-Dendrix analysis was identical to the data input used for NBS analysis, except for genes that shared the same breakpoints, which were now grouped in “pools” (S8 Table). Multi-Dendrix parameters were set to k7t7s11 [15].

## Results

### Detection of recurrent breakpoint genes

Chromosomal CNA status of 352 primary advanced CRC samples was determined using 180K Agilent arrays that cover the genome with an average probe spacing of approximately 17kb, as previously described [9]. Following DNA copy number segmentation, the genomic locations of changes in DNA copy number status were now used to estimate the position of CNA-associated chromosomal breakpoints (Fig 1A and 1B). Statistical evaluation yielded 1605 genomic breakpoint locations with recurrences in multiple CRC samples (FDR<0.1; S1 Table), indicating that the position of CNA-associated breaks is often non-random. When grouping breakpoints by gene affected, a total of 748 recurrent breakpoint genes were identified (FDR<0.1; S2 Table). The genome distribution and prevalence of chromosomal breakpoints at the q-arm of chromosome 13 is provided in Fig 1C and for all other chromosomes in S1 Fig.

**Fig 2:**
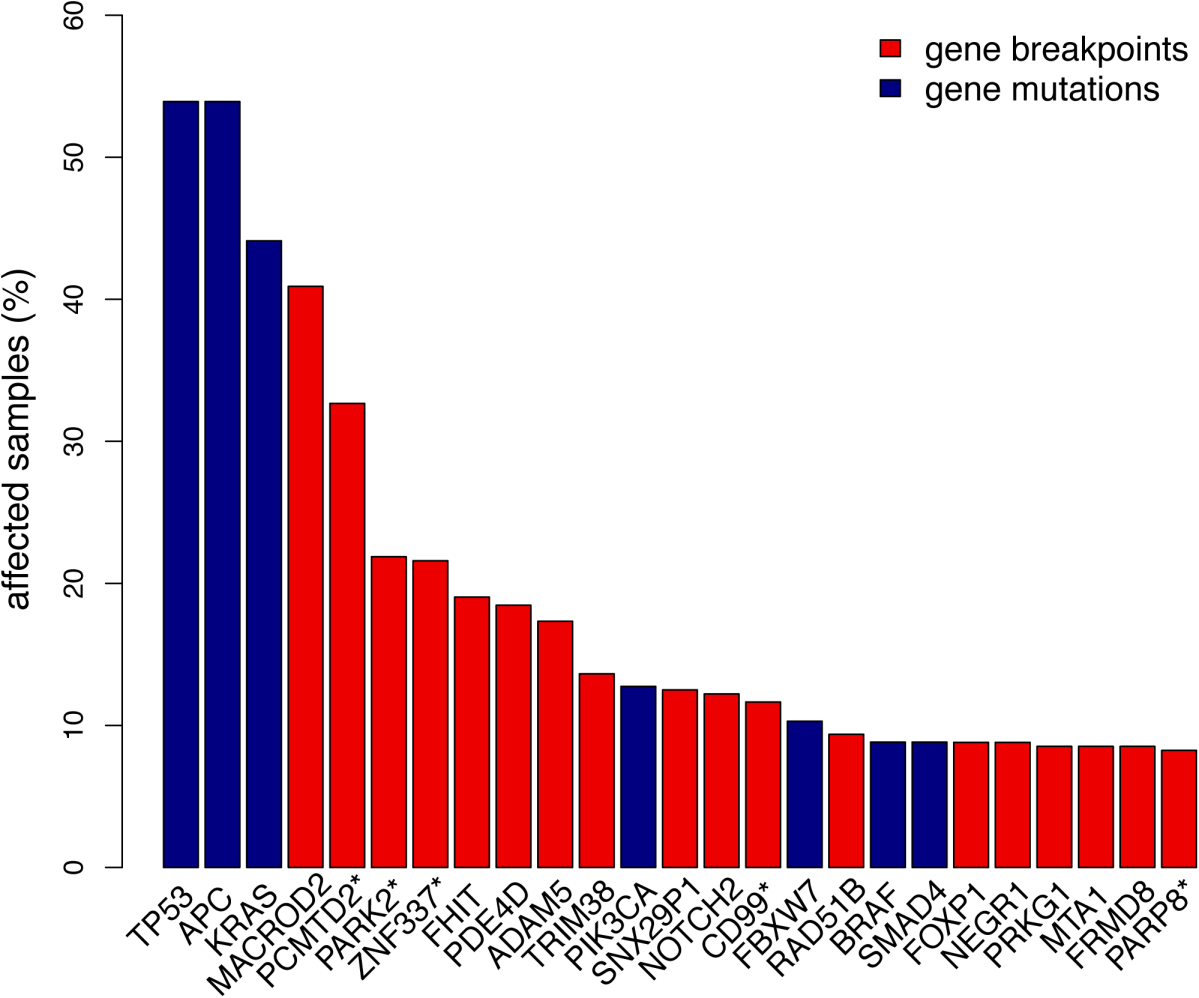
Gene breakpoint and gene mutation frequencies of the 25 most frequently affected genes in CRC. Gene breakpoint frequencies (red bars) were based on the analysis of 352 CRC samples and gene mutation frequencies (blue bars) on the analysis of 204 samples. Genes marked with a “*” indicate a pool of genes that share probe(s) associated with chromosomal breakpoints: the PCMTD2* pool also includes LINC00266-1; PARK2* also includes *PACRG*; ZNF337* also includes *NCOR1P1*, *FAM182A*, *FAM182B*, *FRG1B*, *MIR663A*, *MLLT10P1*; CD99* also includes *XG*; PARP8* also includes *EMB*.

The gene with highest prevalence of chromosomal breakpoints was *MACROD2*, which was affected in 40.9% of CRC samples. Another 169 recurrent breakpoint genes were affected in >3% of advanced CRC samples, similar to the mutation frequencies of commonly affected oncogenes and tumor suppressor genes (Fig 2).

### Clinical relevance of recurrent breakpoint genes

Recurrent breakpoint genes may represent genomic regions that are vulnerable to chromosomal breaks, *i*.*e*. an epiphenomenon associated with CNAs. Alternatively, recurrent breakpoint genes may drive cancer and undergo positive selection during tumorigenesis, and consequently affect clinical outcome such as patient overall survival (OS). Therefore, for each of the recurrent breakpoint genes that was identified, the OS of the subgroup of patients with that specific gene breakpoint was compared to the subgroup of patients without that breakpoint. None of the individual recurrent breakpoint genes was significantly associated with OS (log-rank *P*-values followed by Bonferroni correction for multiple testing, data not shown).

**Fig 3:**
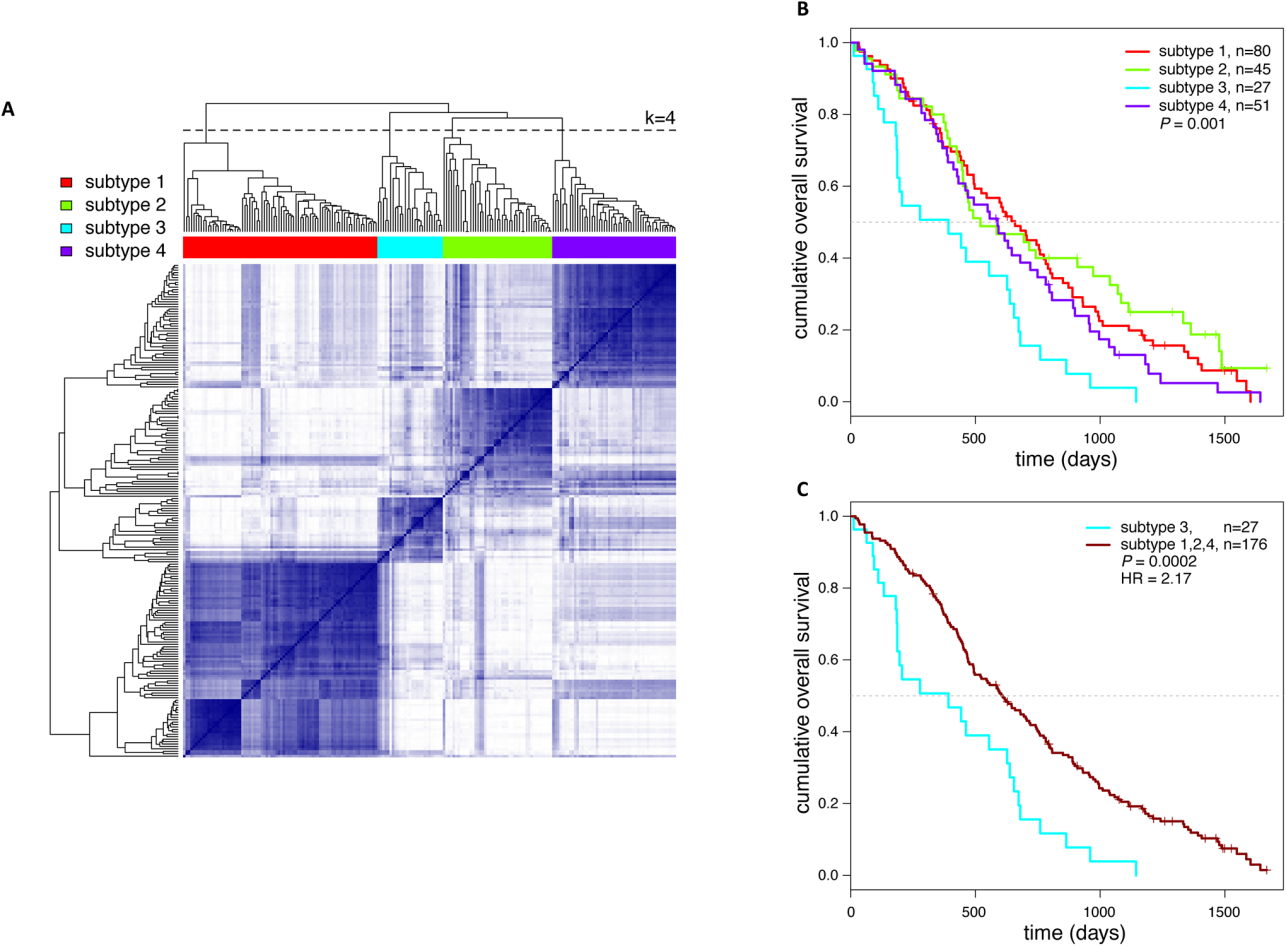
Clustering of 203 CRC patients by NBS based on gene breakpoints and gene mutations revealed four CRC subtypes. (A) Co-clustering matrix of CRC samples generated by NBS analysis. The matrix color intensity represents the similarity score. The color bar on top indicates the groups of patients related to the four CRC subtypes (k = 4) as determined by hierarchical clustering after NBS analysis. (B) Kaplan-Meier plot for overall survival (in days) of CRC subtype 1 (n = 80 patients), subtype 2 (n = 45 patients), subtype 3 (n = 27 patients) and subtype 4 (n = 51 patients). There are significant differences in OS among the four CRC subtypes (log-rank *P* = 0.001), with poorest OS for subtype 3 CRC patients. (C) Kaplan-Meier plot for OS of CRC subtype 3 patients *versus* patients in other CRC subtypes, showing a hazard ratio (HR) of 2.17 and a median OS of 392 days *versus* 610 days, respectively (log-rank *P* = 0.0002).

Cancer-related biological processes are complex and controlled by the concerted action of multiple genes. For that reason we performed a combined analysis of the 170 most prevalent (>3%) recurrent breakpoint genes described above and gene mutation status of key cancer genes. Using DNA isolated from formalin-fixed paraffin-embedded archival tissue, mutation status of *TP53*, *APC*, *KRAS*, *PIK3CA*, *FBXW7*, *SMAD4*, *BRAF* and *NRAS* was determined by targeted next generation sequencing, which succeeded for 204 CRC samples (Fig 2, S3 and S4 Tables). As one case lacked breakpoints and mutations for the selected genes, 203 cases were available to provide both gene breakpoint and gene mutation data as input for Network Based Stratification (NBS) [14]. NBS was applied to propagate sparse gene breakpoint and gene mutation events to the predefined protein interaction network STRING followed by clustering of patients into CRC subtypes based on the affected sub-networks [14]. This analysis revealed four CRC subtypes (Fig 3A and S6 Table). Baseline clinicopathological patient characteristics were highly comparable across the four CRC subtypes (one-side Fisher Exact test; S5 Table). OS analysis revealed significant differences among these subtypes (log-rank *P* = 0.001; Fig 3B), with CRC subtype 3 having a significantly poorer OS than the other three CRC subtypes (HR = 2.17; log-rank *P* = 0.0002; Fig 3C), with 218 days difference in median overall survival.

When further exploring the networks associated with this CRC classification, most of the contributing genes turned out to be recurrent breakpoint genes supplemented with some of the commonly mutated CRC oncogenes and tumor suppressor genes (S7 Table). At an individual gene level within the identified CRC subtype-associated genes, the poor prognostic CRC subtype 3 was *a*.*o*. enriched for gene point mutations in *BRAF* (two-sided Fisher Exact test: *P*<0.0001) and *FBXW7* (*P* = 0.01), and for gene breakpoints in *WWOX* (*P*<0.0001), *FHIT* (*P*<0.0001), and *PIBF1* (*P* = 0.03). Because mutations in *BRAF* are often associated with microsatellite instable (MSI) tumors [16], we examined the distribution of MSI samples across the four CRC subtypes. Interestingly, eight out of ten MSI samples in this group of 203 CRCs were in subtype 3 (two-sided Fisher Exact test: *P*<0.0001; S5 Table). Taken together, these data indicate that CNA-associated recurrent breakpoint genes are clinically relevant as they contribute to CRC classification of subtypes with prognostic value.

### Biological relevance of recurrent breakpoint genes

**Fig 4:**
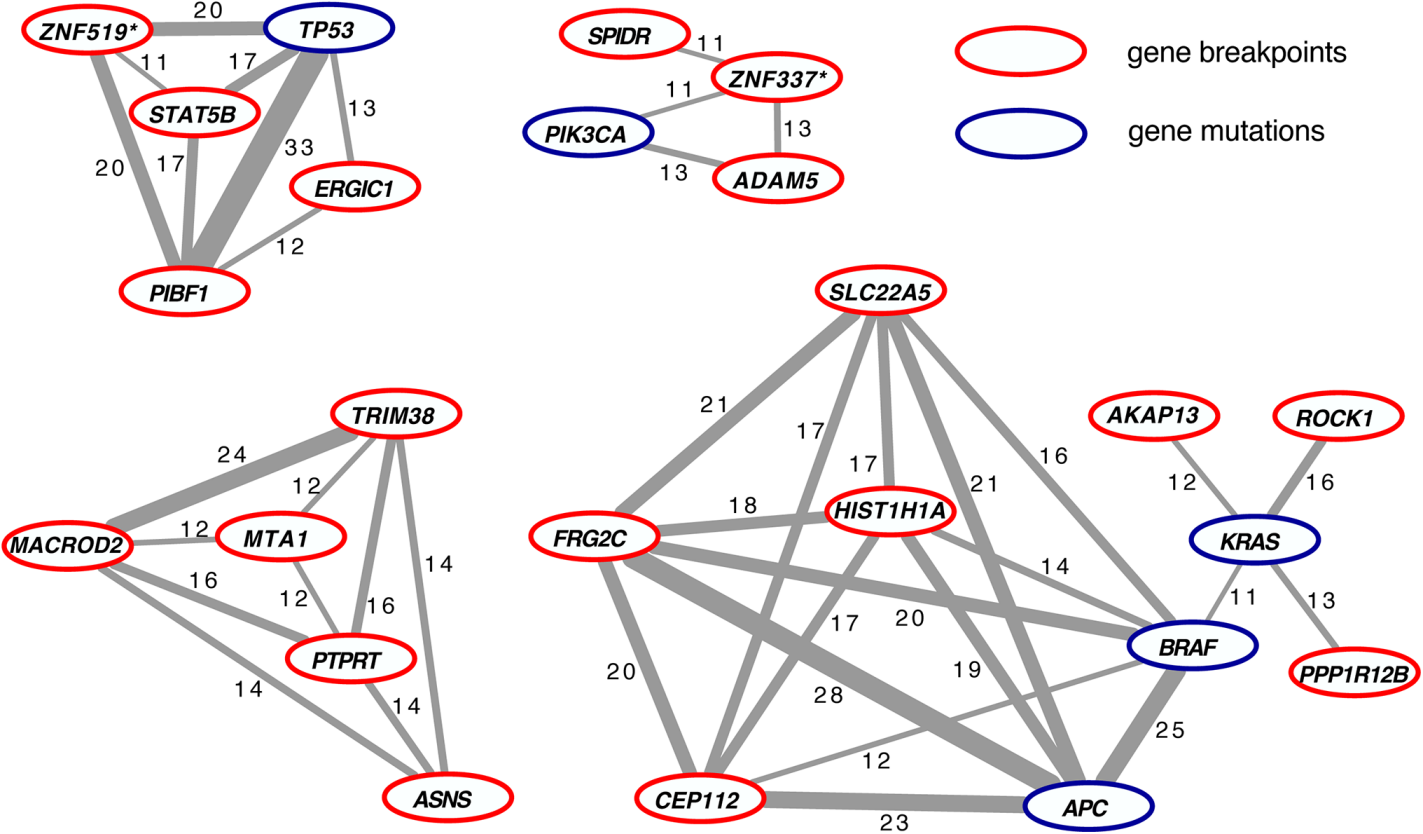
Four distinct core modules of putative CRC driver genes were retrieved by Multi-Dendrix analysis. The nodes comprise both gene breakpoints (red outline) and gene mutations (blue outline). Edges (grey lines) connect genes that are mutually exclusively affected. The thickness of the grey lines and the corresponding number reflect the robustness score. The strongest mutual exclusivity is observed between *PIBF1* and *TP53*. Genes marked with a “*” indicate a pool of genes that share probe(s) associated with chromosomal breakpoints: the ZNF337* pool also includes *NCOR1P1*, *FAM182A*, *FAM182B*, *FRG1B*, *MIR663A*, *MLLT10P1*; ZNF519* also includes *ANKRD20A5P*, *ANKRD30B*.

To further investigate whether recurrent breakpoint genes may drive CRC development, the Multi-Dendrix algorithm was applied to identify oncogenic pathways or gene modules based on two criteria, namely: 1) the events within one module must be mutually exclusive, and 2) these events should cover nearly all cancer samples studied [15]. The input data for this analysis was identical to that for NBS, *i*.*e*. the gene mutation status of eight commonly affected CRC genes and the breakpoint status of the 170 most prevalent (>3%) recurrent breakpoint genes from 203 CRC samples. This analysis revealed four distinct gene modules, three modules containing both gene mutations and gene breakpoints and one module being entirely composed of recurrent breakpoint genes (Fig 4). The strongest mutual exclusivity was observed between *TP53* mutations and *PIBF1* breakpoints, a gene whose breakpoints were most prevalent in the CRC subtype 3 that showed poorest prognosis. The genomic location of *PIBF1* on the q-arm of chromosome 13 is highlighted in Fig 1D, illustrating enrichment of gene breakpoints in the distal part of this gene. Moreover, also *MACROD2*, *PPP1R12B*, *AKAP13*, *ERGIC1*, *PTPRT*, *SLC22A5*, *HIST1H1A*, *ASNS*, and *ROCK1* breakpoint genes were represented in one of the gene modules, and contributed to CRC subtype classification (Fig 4 and S7 Table). These data imply that multiple recurrent breakpoint genes play an important biological role in CRC development.

## Discussion

Molecular characterization of somatic DNA aberrations is a helpful strategy to aid prognosis and therapy prediction of individual patients. While the analysis of non-synonymous point mutations in commonly mutated cancer genes and determination of chromosomal CNAs have become standard of practice for characterizing tumor samples, large-scale genome-wide detailed analysis of SVs is still in its infancy. We here demonstrated that CNA profiles allow to detect genes whose function may be affected by CNA-associated chromosomal breaks. In total 748 recurrent breakpoint genes were identified based on the analysis of a large series (n = 352) of high-resolution array-CGH samples of primary tumors from patients who participated in two phase III clinical trials in metastatic CRC. In addition to their abundance also the prevalence of recurrent breakpoint genes was relatively high, with 170 genes being affected by chromosomal breaks in more than 3% of cancer cases. As such, the prevalence of genes affected by CNA-associated chromosomal breaks is comparable to the prevalence of point mutations in well-known and commonly affected CRC oncogenes and tumor suppressor genes (Fig 2).

One of the key questions we aimed to address is whether chromosomal breaks within genes are just an epiphenomenon associated with chromosomal instability or whether recurrent breakpoint genes represent cancer drivers with biological and clinical relevance. While none of the individual recurrent breakpoint genes exhibited significant effects on OS, propagation of the 170 most prevalent breakpoint genes in combination with eight commonly mutated genes onto a predefined network allowed to classify CRC into four subtypes by NBS. One of the CRC subtypes, CRC subtype 3, had a significantly poorer prognosis than the others (Fig 3), indicating that clinically distinct subtypes could be identified. In a multivariate analysis (data not shown) the factors ‘WHO performance status’, ‘LDH at randomization’, ‘prior adjuvant therapy’, ‘tumor stage primary tumor’, ‘number of affected organs’ and ‘MSI status’ were retained. Because genomic mutations are causal for tumorigenesis and dictate tumor behavior, it is very well possible that phenotypic factors, which ultimately are a result of underlying biology, mask the prognostic effect of the genomic CRC subtypes. Such a dependency between clinicopathological prognostic parameters and the CRC subtypes as described here therefore does not refute univariate prognostic value of this classification.

CRC subtype 3 turned out to be enriched for tumors with *BRAF* mutations (33% of cases *versus* 5% in the other CRC samples; S7 Table) and MSI tumors (30% of cases *versus* 1% in the other CRC samples). *BRAF* is mutated at much higher frequencies in MSI tumors than in chromosomal instable tumors, and within MSI tumors, *BRAF* mutation is known to be associated with poor prognosis [16]. Although MSI tumors have a relatively good prognosis in early stage disease, they also are associated with explicitly poor prognosis in metastatic CRC [17]. MSI tumors often have a lower frequency of CNA aberrations than tumors that are microsatellite stable (MSS) and therefore have less CNA-associated chromosomal breakpoints than MSS tumors. This suggests that MSI tumors may become clustered into one distinct CRC subtype irrespective of (alterations in) function of recurrent breakpoint genes. Following this line of reasoning, one would predict that the poor prognosis CRC subtype 3 lacks recurrent breakpoint genes with increased mutation frequencies compared to the other CRC subtypes. However, our data showed otherwise (S7 Table), with significant enrichment of breakpoint frequencies in CRC subtype 3 *versus* the other CRC samples for *WWOX* (33% *versus* 5%), *FHIT* (59% *versus* 13%), and *PIBF1* (15% *versus* 3%). These data emphasize that recurrent breakpoint genes contribute significantly to clinically relevant CRC classification.

As our data support clinical relevance of recurrent breakpoint genes, it is expected that chromosomal breaks within these genes somehow result in positive selection of cancer cells and stimulate tumor development. Functional analysis of recurrent breakpoint genes to understand their biological effects was beyond the scope of the current study. However, for many of these a role in tumorigenesis has been described in literature. *WWOX* and *FHIT* have long been known to reside at common fragile sites and have been demonstrated to act as suppressors of tumor development by gene knockout mouse models [18]. Moreover, *WWOX* overexpression was shown to promote the immune response in a glioma model [19] while *FHIT* positively regulates expression of MHC class I molecules on cancer cells [20]. These data suggest that loss of function of *WWOX* and *FHIT* help to escape immunosurveillance. Likewise, the progesterone immunomodulatory binding factor *PIBF1* was identified as a secreted factor that can prevent pregnancy loss by dampening the immune response. Considering that *PIBF1* breakpoints were predominantly observed in the distal part of the gene (Fig 1D) it is tempting to speculate that *PIBF1* gene breakpoints disrupt its nuclear localization signal upon which it becomes a secreted protein with anti-tumor immune-suppressing capabilities [21]. MSI tumors are thought to evoke an anti-tumor immune response that prevents metastatic spread, however, once circumvented these tumors become very aggressive [16]. In this respect it is of interest to note that the breakpoint genes that contributed most to the classification of the poor prognosis CRC subtype 3, *i*.*e*. *WWOX*, *FHIT*, and *PIBF1*, all have been implicated to modulate immune responses.

*MACROD2* was the most prevalent recurrent breakpoint gene in our cohort, being affected in 41% of CRC cases. This gene was also one of the most frequently observed rearranged genes through a focal deletion in other studies [3,22]. *MACROD2* is able to hydrolyze endogenous mono-ADP-ribosyl groups, a reversible post-translational modification moiety, from target proteins such as *GSK3B*. This restores the function of *GSK3B*, which is a key inhibitor of the Wnt signaling pathway. Hence, the absence of functional *MACROD2* may decrease the kinase activity of *GSK3B* and thereby promote Wnt signaling [23–25]. Moreover, *MACROD2* may play a role in modulating the function of histone proteins and is recruited in case of DNA damage response [23,25].

To further address the biological relevance of recurrent breakpoint genes we tried to construct modules of putative cancer driving genes, using Multi-Dendrix. On the one hand, this analysis can reveal oncogenic pathways by looking for mutually exclusive gene mutation patterns that cover nearly all CRC samples. On the other hand, if an apparently homogeneous group of tumors consists of distinct tumor subtypes, mutual exclusivity between genes can also reflect the presence of (previously unrecognized) cancer subtypes. The strongest mutual exclusivity was observed between *TP53* mutations and *PIBF1* breakpoints (Fig 4). Loss of *TP53* function is a critical step towards chromosomal instability while *PIBF1* breakpoints were enriched in CRC subtype 3, which harbored the majority of MSI tumors. Therefore, the *TP53*—*PIBF1* module may represent genes that drive genomic instability, comprising distinct chromosomal instable and microsatellite instable CRC subtypes. Another gene module contained *APC*, *KRAS* and *BRAF* mutations, *i*.*e*. somatic alterations that are known to occur early in tumor development compared to *TP53* aberrations. This module supports the known mutual exclusivity between *KRAS* and *BRAF* mutations. Moreover, it included *ROCK1* breakpoints, which are enriched in CRC subtype 4 (S7 Table). Interestingly, inhibitors of *ROCK1* improve the success rate of bringing embryonic stem cells into culture [26] and are supplemented to the medium for *in vitro* cultures of human colon epithelial organoids, indicating that inhibition of *ROCK1* supports early stages of tumor development. With the exception of *ROCK1*, there appeared to be limited overlap between recurrent breakpoint genes in the Multi-Dendrix modules and the currently well-studied signal transduction pathways in cancer, suggesting that recurrent breakpoint genes may affect carcinogenesis through mechanisms that require further research.

*MACROD2* was part of a module together with four other recurrent breakpoint genes, *i*.*e*. *TRIM38*, *MTA1*, *PTPRT* and *ASNS*. The fact that these genes appear in one module suggests that they may act together in one pathway. Although extensive knowledge about the function of these genes is not available, it appears that most of them are (in)directly capable to affect Wnt signaling. As discussed, loss of *MACROD2* can promote Wnt signaling through decreasing *GSK3B* kinase activity [23–25]. Also *MTA1* has been described to affect Wnt signaling through modulation of *GSK3B* activity [27]. *PTPRT* is able to dephosphorylate *STAT3* [28], which in turn can interact with and modulate the function of the WNT pathway mediator β-catenin [29]. The canonical Wnt pathway may also be activated by elevation of NF-κB signaling, which results in cell dedifferentiation towards tumor-initiating cells [30]. *TRIM38* may counteract NF-κB activity [31], suggesting that loss of *TRIM38* might enhance tumorigenesis. Taken together, this module comprises genes whose function may directly or indirectly affect different aspects of the Wnt pathway, a hypothesis that needs further investigation by functional analysis of (combinations of) these genes.

Technically, the genome-wide detection of SVs at nucleotide resolution is currently possible by next generation sequencing. However, in practice genome-wide analyses of SVs of large sample series with well-documented patient survival and other clinical information are scarce. In contrast, DNA CNA profiles of primary tumors are abundantly available in public archives and are still being generated for molecular characterization of cancer samples. In our study, the molecular profile of the primary tumors was used to characterize metastatic disease, because DNA aberrations of metastases show high concordance with the patient-matched primary tumor counterpart [32,33]. We now demonstrated that these commonly and widely available DNA CNA profiles allow detection of a significant subset of SVs, *i*.*e*. genes with CNA-associated chromosomal breaks. Importantly, we showed that recurrent breakpoint genes are highly prevalent and clinically relevant, emphasizing the need to characterize larger sample series from more tumor types to fully appreciate their impact. We therefore argue that, in addition to gene mutation and gene copy number analyses, molecular characterization of cancer samples should also comprise the detection of recurrent breakpoint genes.

## Supporting Information

S1 Fig Chromosomal breakpoint frequencies and their distribution over chromosomes. (PDF)

S1 Table Chromosomal breakpoints, probe-level. (XLSX)

S2 Table Chromosomal breakpoints, gene-level. (XLSX)

S3 Table Gene mutation matrix, per CRC sample. (XLSX)

S4 Table Gene mutation frequencies, summary. (XLSX)

S5 Table Baseline patient characteristics. (XLSX)

S6 Table CRC subtype and OS data. (XLSX)

S7 Table CRC subtype-associated genes. (XLSX)

S8 Table Pools of genes that share probe(s) associated with chromosomal breakpoints. (XLSX)
