# Supplementary material for: High Prevalence and Clinical Relevance of Genes Affected by Chromosomal Breaks in Colorectal Cancer
Source: PLoS One. 2015 Sep 16;10(9):e0138141. doi: 10.1371/journal.pone.0138141 (PMC4574474; doi:10.1371/journal.pone.0138141)
Supplement: S1 Fig — (PDF) [file pone.0138141.s001.pdf]

Supplementary Figure S1

Graphical representation of CNA-associated chromosomal breakpoint frequencies and their distribution over chromosomes 1-22 and X (numbered 23). The X-axes depict the genomic position in Mb. The Y-axes depict the chromosomal breakpoint frequencies across the cohort of 352 CRC samples. Breakpoint frequencies are indicated on array-CGH probe-level (vertical black bars) and on gene-level (horizontal red bars). Recurrent breakpoint genes (FDR<0.1) are named. When the gene breakpoint frequency exceeded 10% (horizontal dashed line), the breakpoint frequency (%) follows the gene name.

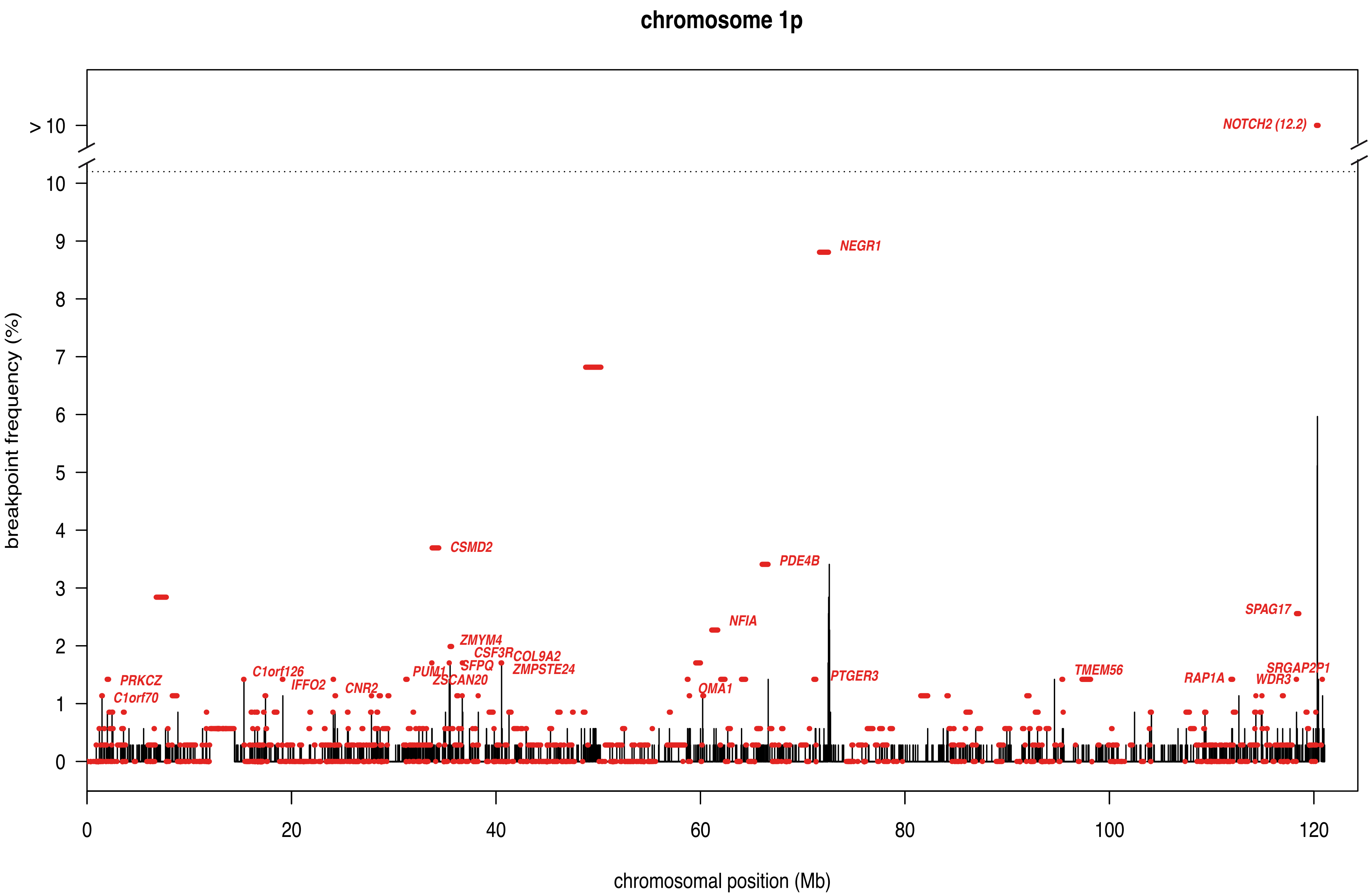
chromosome 1p
breakpoint frequency (%)
chromosomal position (Mb)
> 10
NOTCH2 (12.2)
NEGR1
CSMD2
PDE4B
NFIA
SPAG17
ZMYM4
CSF3R
COL9A2
ZMPSTE24
SFPQ
PUM1
ZSCAN20
C1orf126
IFFO2
CNR2
PRKCZ
C1orf70
OMA1
PTGER3
TMEM56
RAP1A
WDR3
SRGAP2P1
0
1
2
3
4
5
6
7
8
9
10
0
20
40
60
80
100
120

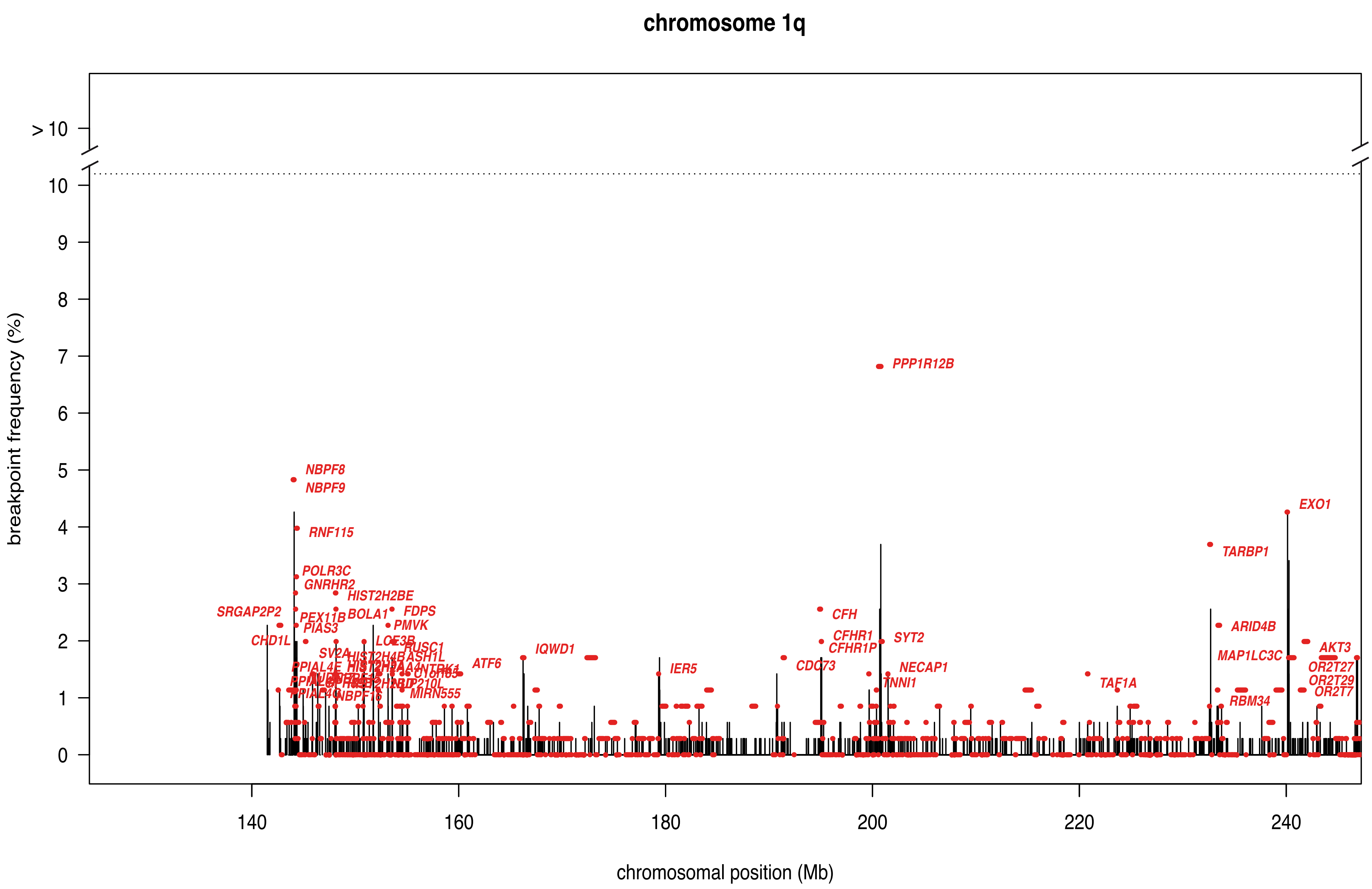
chromosome 1q
breakpoint frequency (%)
chromosomal position (Mb)
> 10
10
9
8
7
6
5
4
3
2
1
0
140
160
180
200
220
240
PPP1R12B
NBPF8
NBPF9
RNF115
POLR3C
GNRHR2
HIST2H2BE
SRGAP2P2
PEX11B
BOLA1
FDPS
PIAS3
PMVK
CHD1L
SV2A
ATF6
IQWD1
IER5
CFH
CFHR1
CFHR1P
SYT2
CDC73
NECAP1
TNNI1
TAF1A
EXO1
TARBP1
ARID4B
MAP1LC3C
RBM34
AKT3
OR2T27
OR2T29
OR2T7
MIRN555
NBPF16
NTRK1

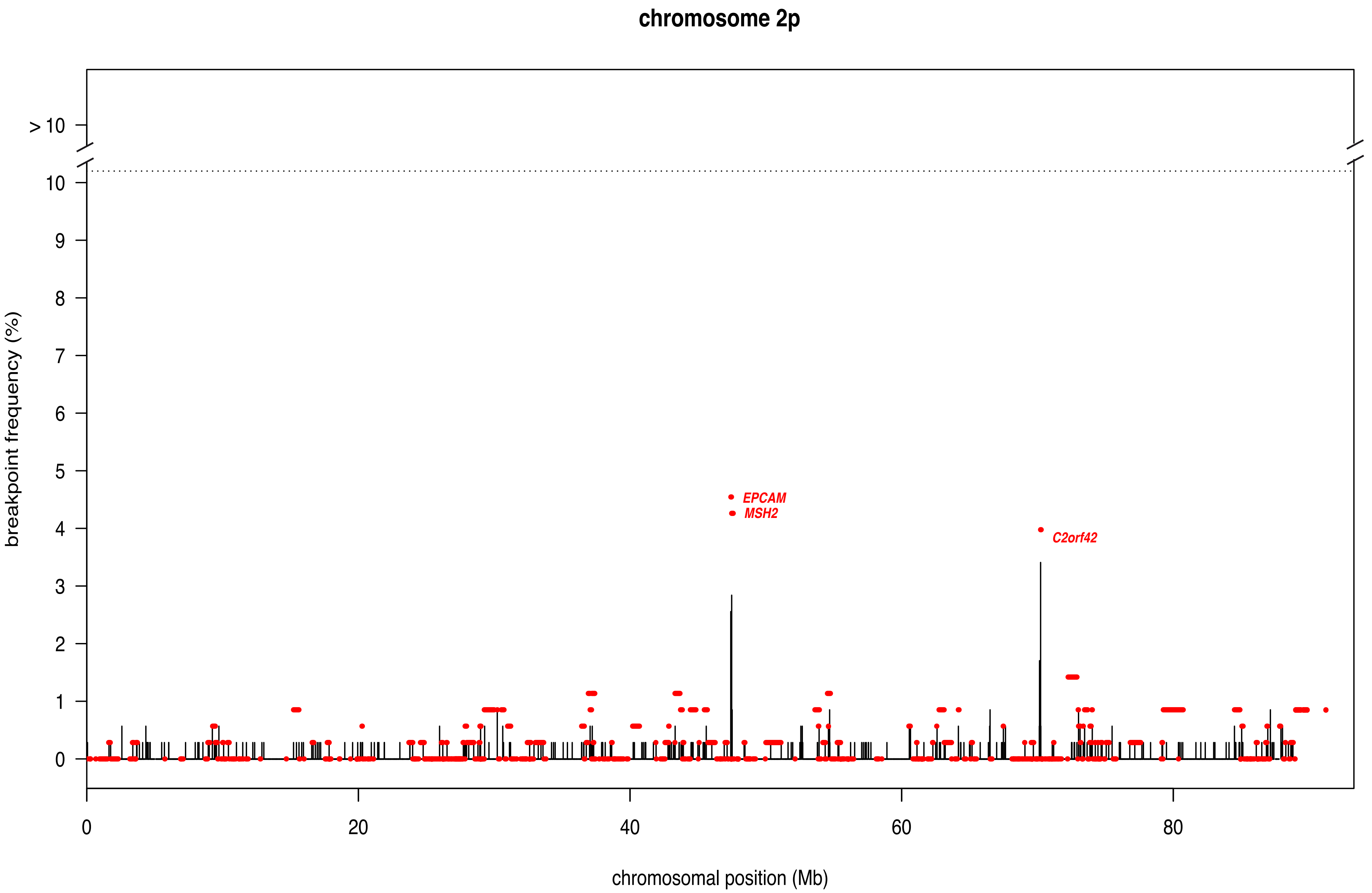

chromosome 2p
breakpoint frequency (%)
chromosomal position (Mb)
> 10
10
9
8
7
6
5
4
3
2
1
0
0
20
40
60
80
EPCAM
MSH2
C2orf42

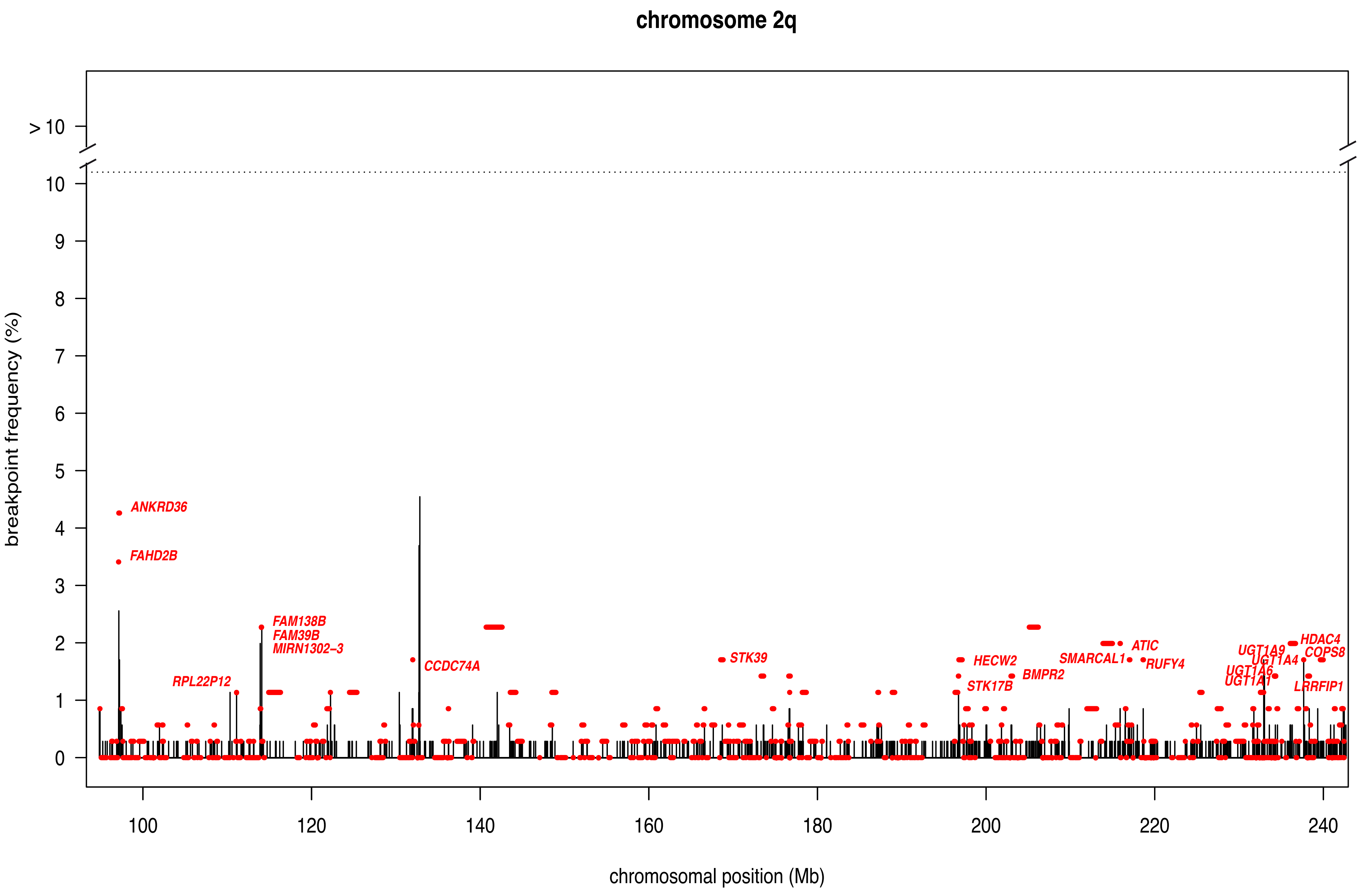
chromosome 2q
breakpoint frequency (%)
> 10
10
9
8
7
6
5
4
3
2
1
0
ANKRD36
FAHD2B
FAM138B
FAM39B
MIRN1302–3
RPL22P12
CCDC74A
STK39
HECW2
STK17B
BMPR2
SMARCAL1
ATIC
RUFY4
UGT1A9
UGT1A4
UGT1A6
UGT1A1
HDAC4
COPS8
LRRFIP1
100
120
140
160
180
200
220
240
chromosomal position (Mb)

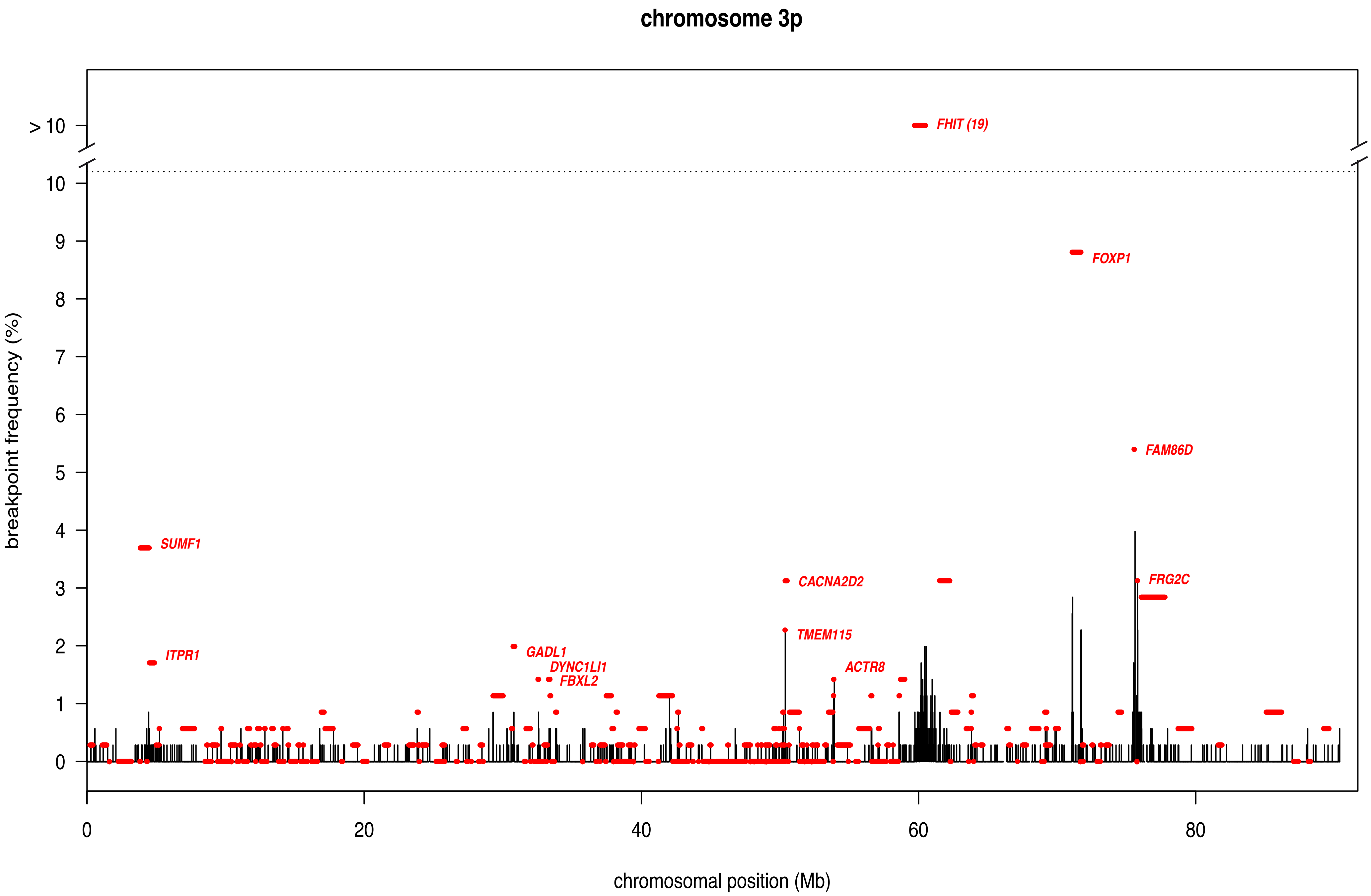
chromosome 3p
FHIT (19)
FOXP1
FAM86D
SUMF1
CACNA2D2
FRG2C
TMEM115
ITPR1
GADL1
DYNC1LI1
FBXL2
ACTR8
breakpoint frequency (%)
chromosomal position (Mb)
> 10
10
9
8
7
6
5
4
3
2
1
0
20
40
60
80

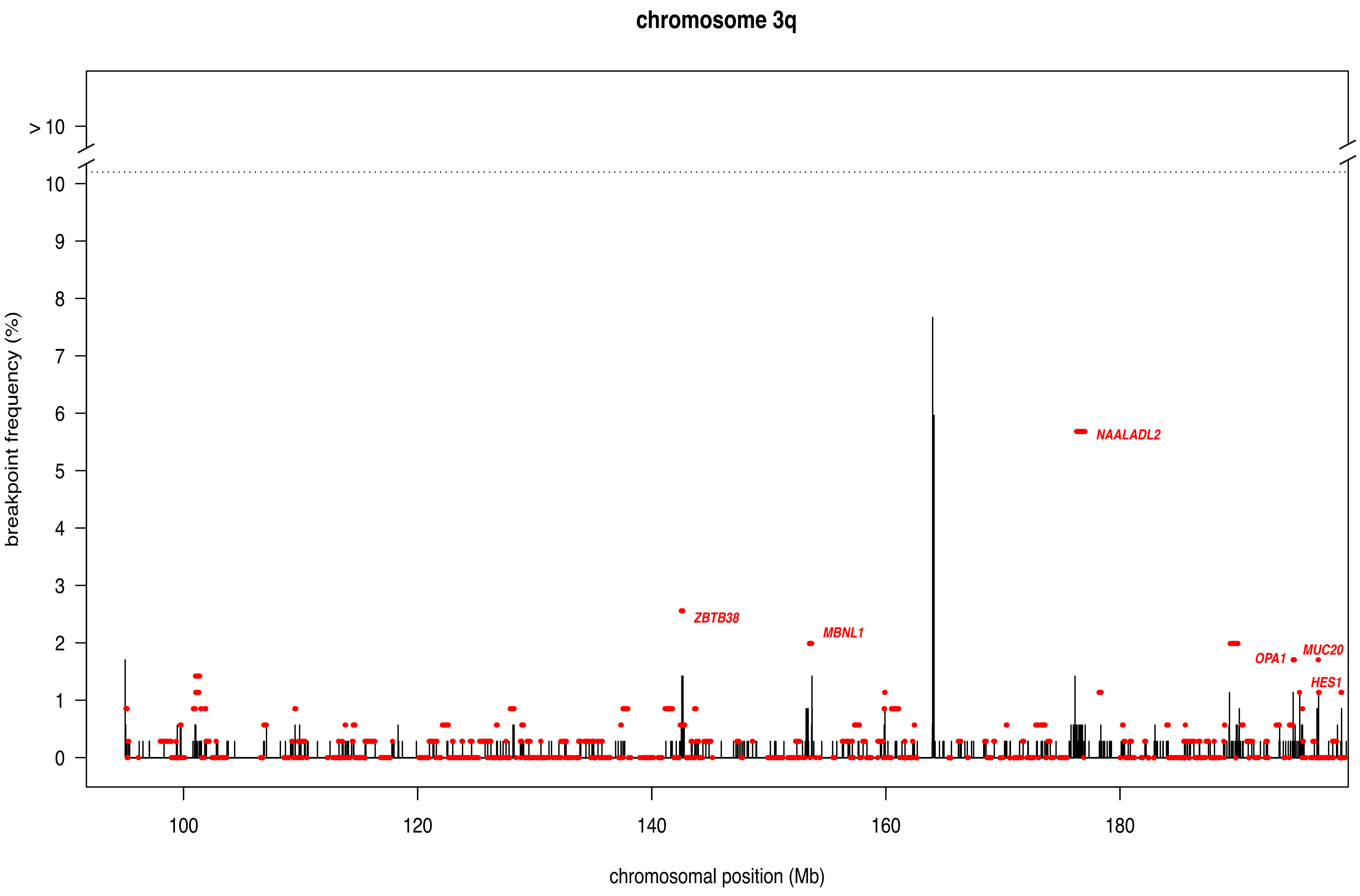

chromosome 3q
breakpoint frequency (%)
chromosomal position (Mb)
> 10
10
9
8
7
6
5
4
3
2
1
0
100
120
140
160
180
NAALADL2
ZBTB38
MBNL1
OPA1
MUC20
HES1

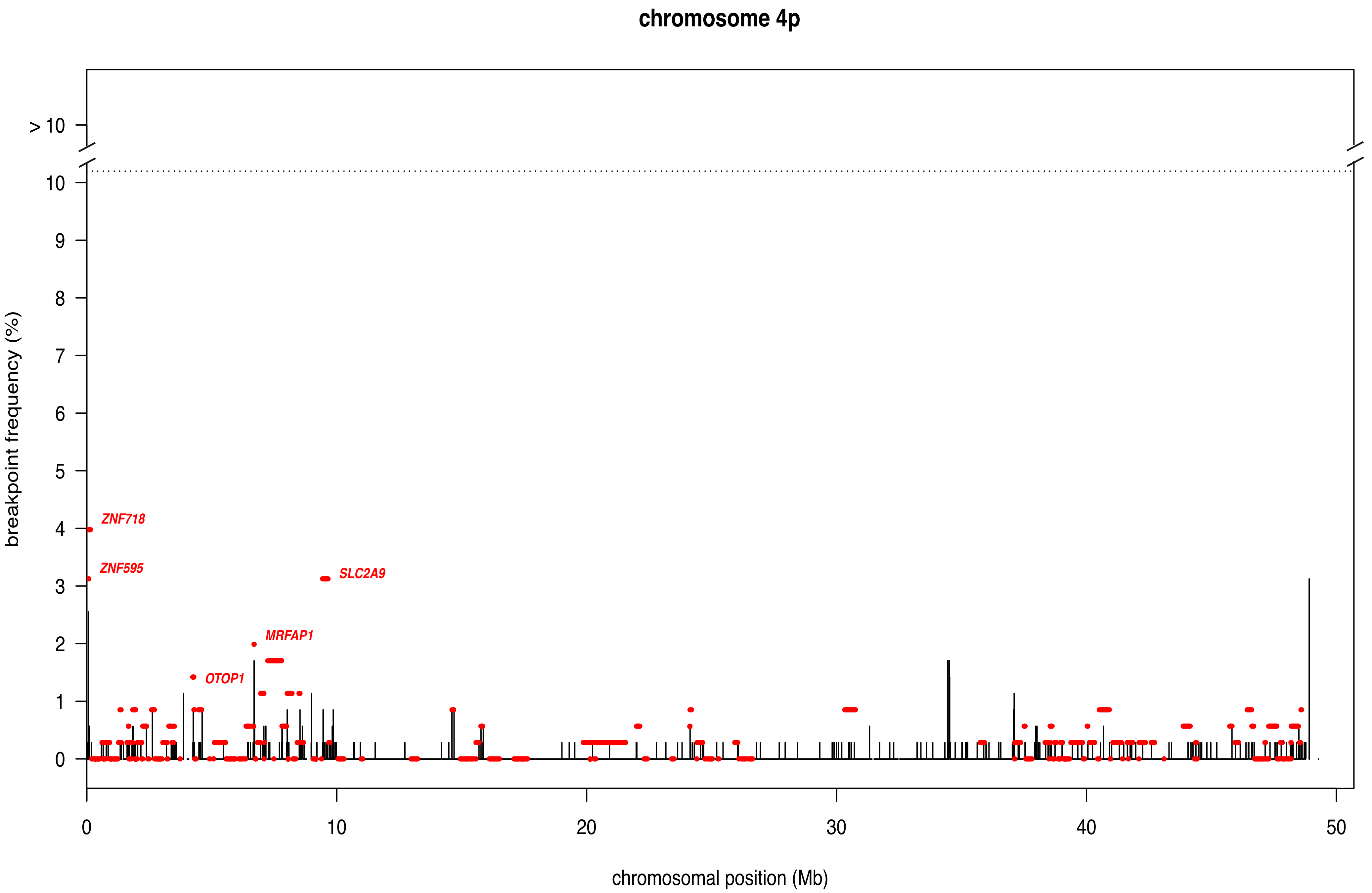

chromosome 4p
breakpoint frequency (%)
chromosomal position (Mb)
> 10
10
9
8
7
6
5
4
3
2
1
0
0
10
20
30
40
50
ZNF718
ZNF595
SLC2A9
MRFAP1
OTOP1

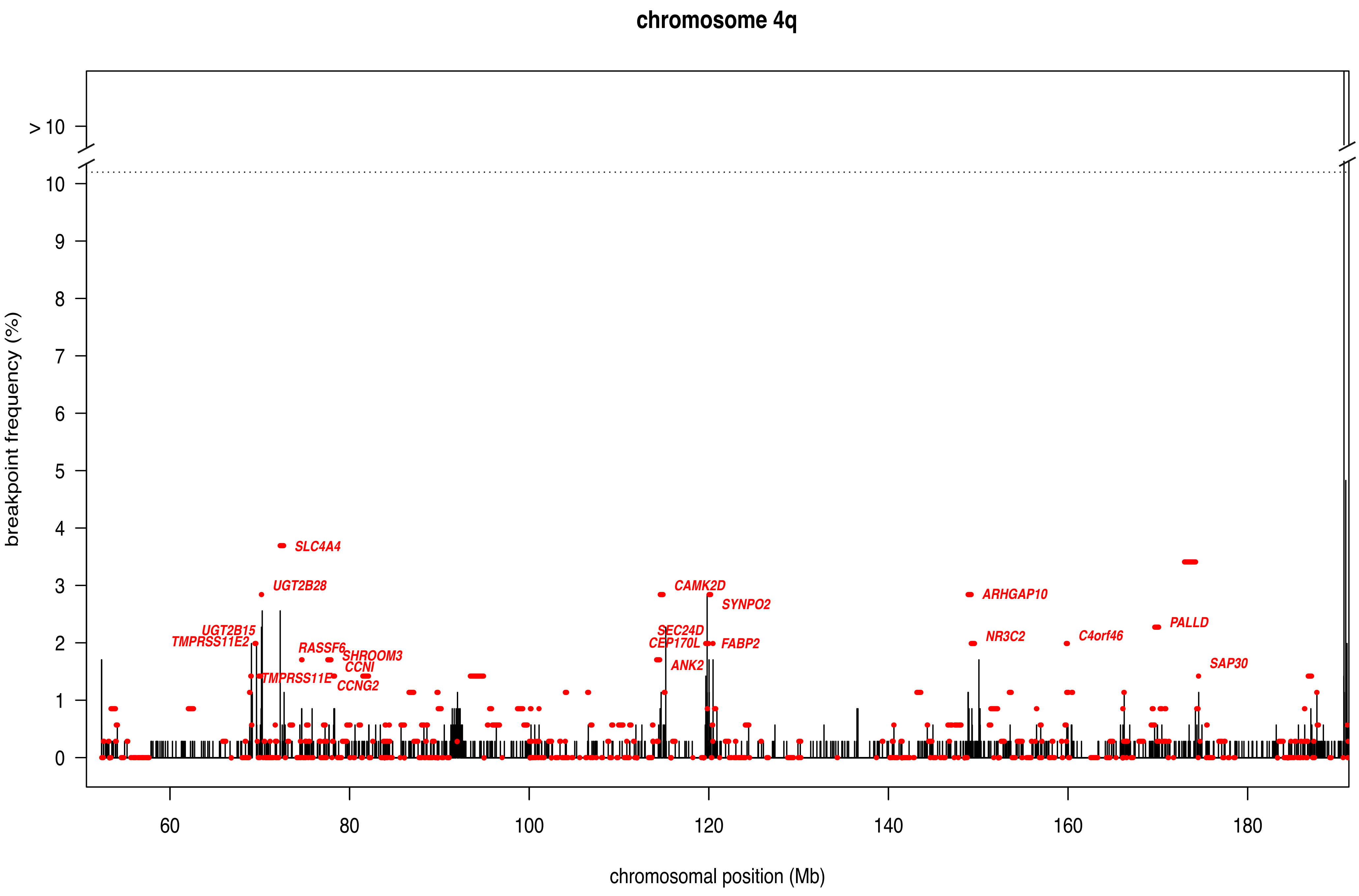

chromosome 4q
breakpoint frequency (%)
chromosomal position (Mb)
> 10
0
1
2
3
4
5
6
7
8
9
10
60
80
100
120
140
160
180
SLC4A4
UGT2B28
UGT2B15
TMPRSS11E2
TMPRSS11E
RASSF6
SHROOM3
CCNI
CCNG2
CAMK2D
SYNPO2
SEC24D
CEP170L
FABP2
ANK2
ARHGAP10
NR3C2
C4orf46
PALLD
SAP30

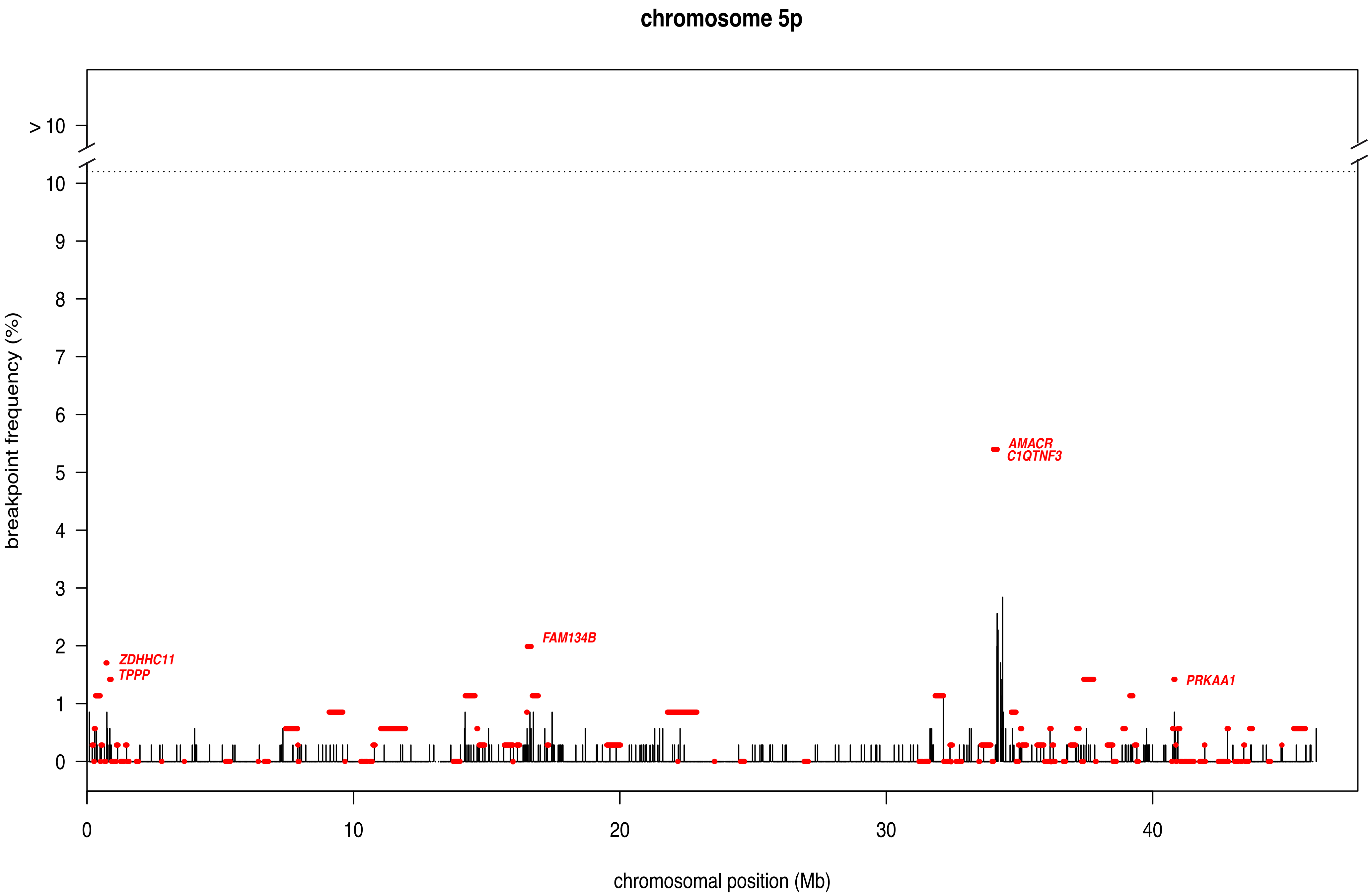
chromosome 5p
breakpoint frequency (%)
chromosomal position (Mb)
> 10
10
9
8
7
6
5
4
3
2
1
0
0
10
20
30
40
AMACR
C1QTNF3
FAM134B
ZDHHC11
TPPP
PRKAA1

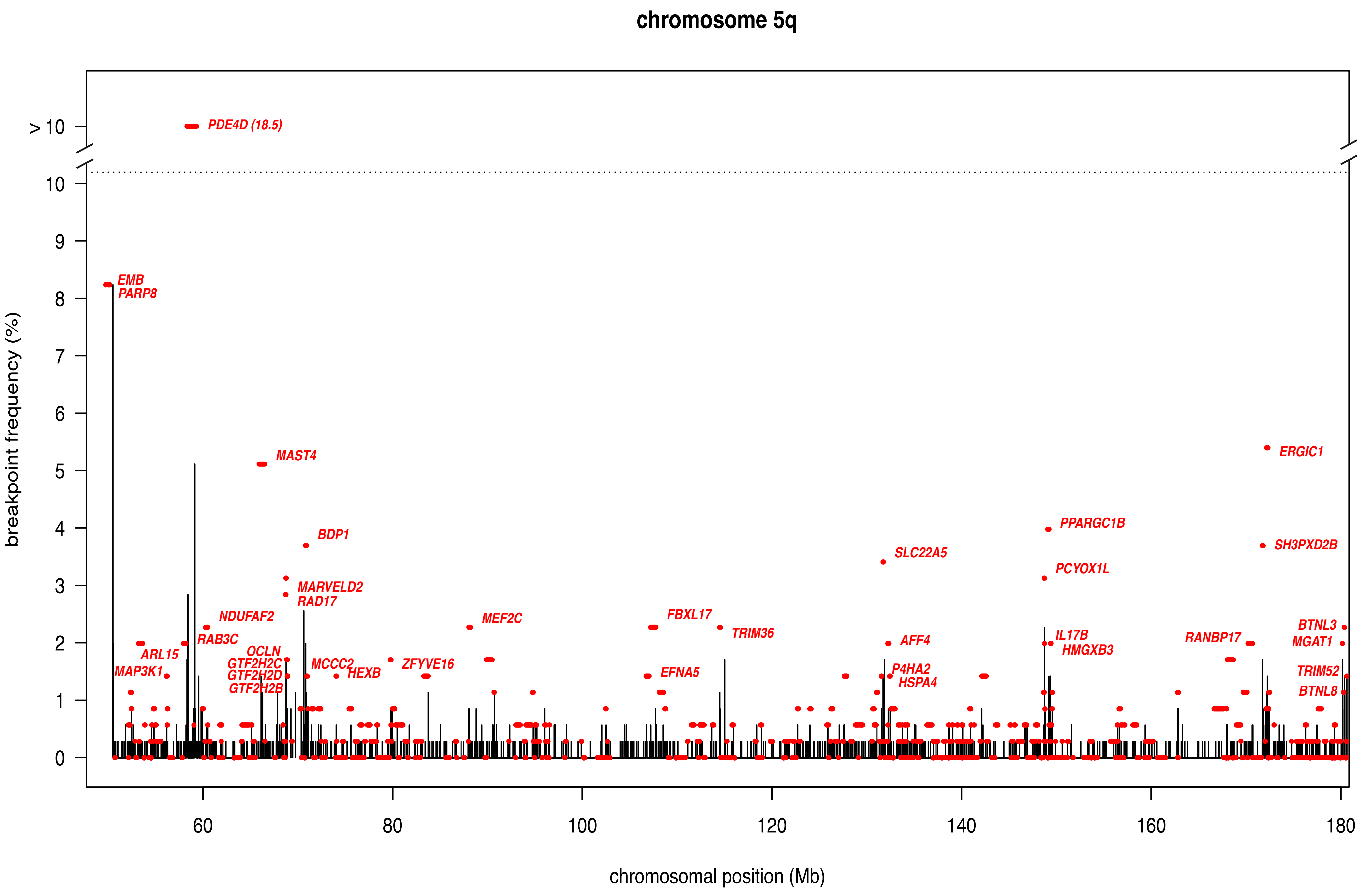
chromosome 5q
breakpoint frequency (%)
chromosomal position (Mb)
> 10
PDE4D (18.5)
EMB
PARP8
MAST4
BDP1
MARVELD2
RAD17
NDUFAF2
RAB3C
ARL15
MAP3K1
OCLN
GTF2H2C
GTF2H2D
GTF2H2B
MCCC2
HEXB
ZFYVE16
MEF2C
FBXL17
TRIM36
EFNA5
SLC22A5
AFF4
P4HA2
HSPA4
PPARGC1B
PCYOX1L
IL17B
HMGXB3
RANBP17
ERGIC1
SH3PXD2B
BTNL3
MGAT1
TRIM52
BTNL8

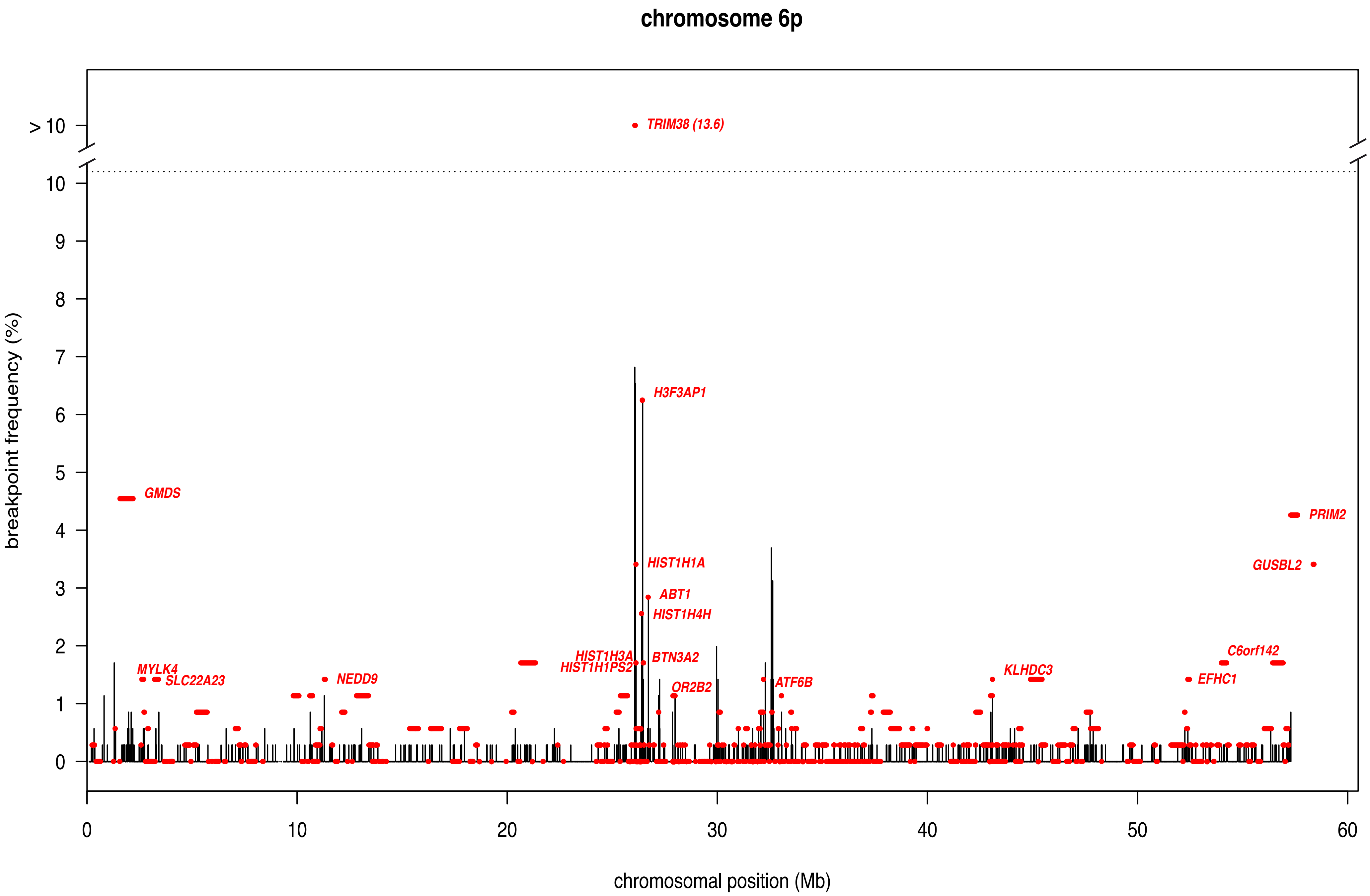
chromosome 6p
breakpoint frequency (%)
chromosomal position (Mb)
> 10
TRIM38 (13.6)
H3F3AP1
GMDS
PRIM2
GUSBL2
HIST1H1A
ABT1
HIST1H4H
HIST1H3A
HIST1H1PS2
BTN3A2
C6orf142
MYLK4
SLC22A23
NEDD9
OR2B2
ATF6B
KLHDC3
EFHC1
0
1
2
3
4
5
6
7
8
9
10
0
10
20
30
40
50
60

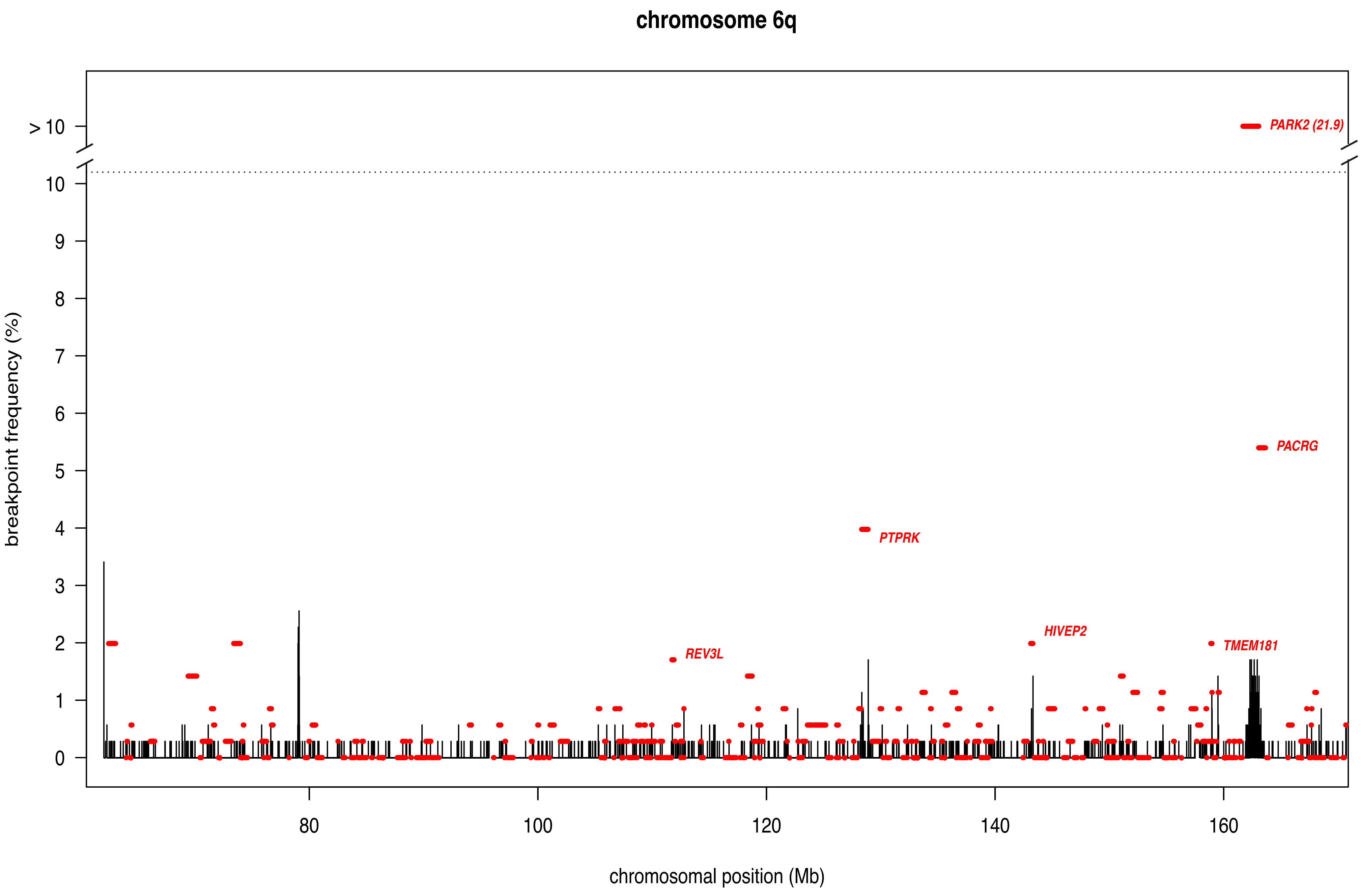

chromosome 6q
> 10
PARK2 (21.9)
PACRG
PTPRK
REV3L
HIVEP2
TMEM181
breakpoint frequency (%)
0
1
2
3
4
5
6
7
8
9
10
80
100
120
140
160
chromosomal position (Mb)

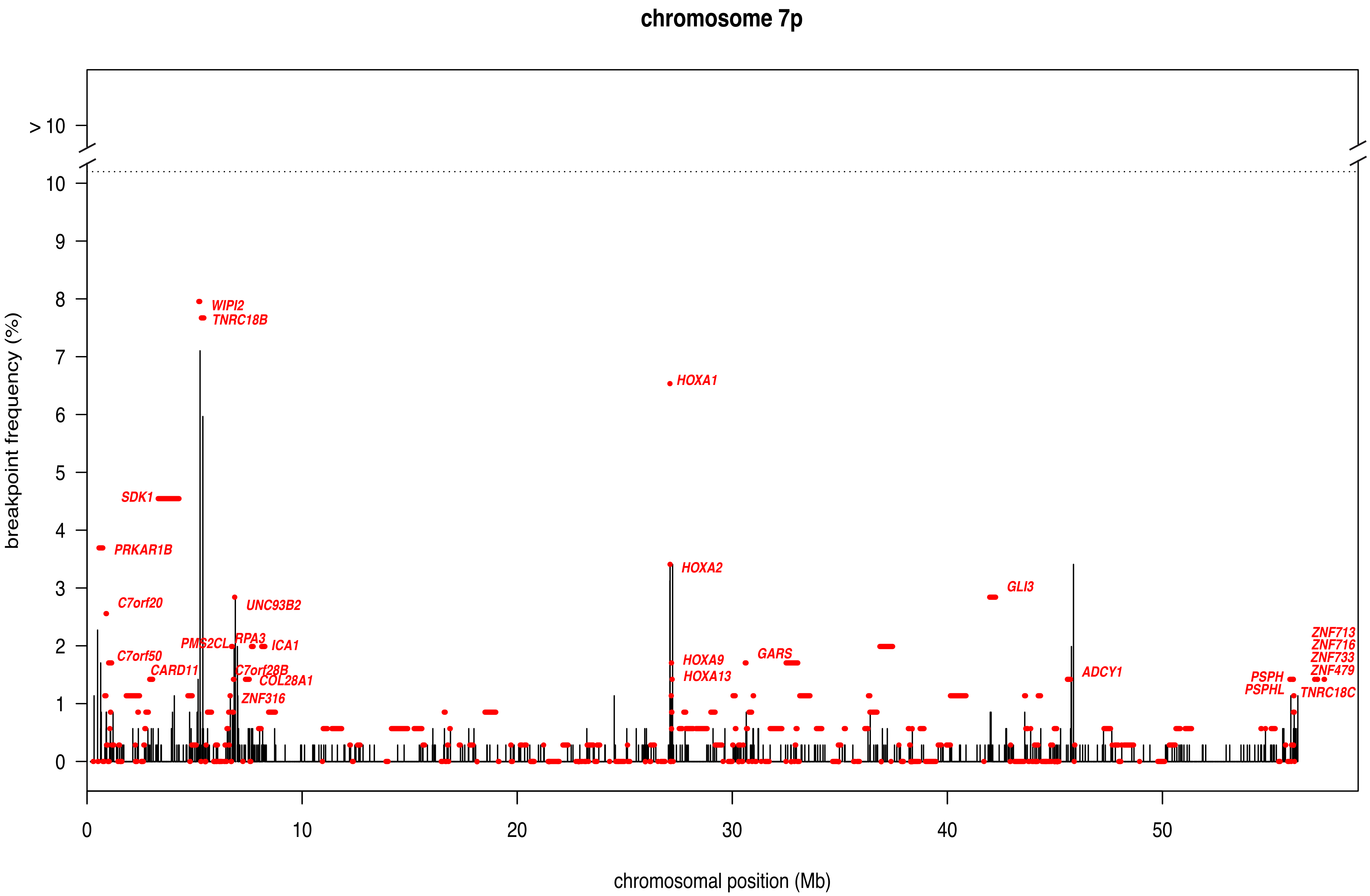

chromosome 7p
breakpoint frequency (%)
chromosomal position (Mb)
> 10
10
9
8
7
6
5
4
3
2
1
0
10
20
30
40
50
WIPI2
TNRC18B
HOXA1
SDK1
PRKAR1B
HOXA2
GLI3
C7orf20
UNC93B2
PMS2CL
RPA3
ICA1
C7orf50
CARD11
C7orf28B
COL28A1
ZNF316
HOXA9
HOXA13
GARS
ADCY1
PSPH
PSPHL
ZNF713
ZNF716
ZNF733
ZNF479
TNRC18C

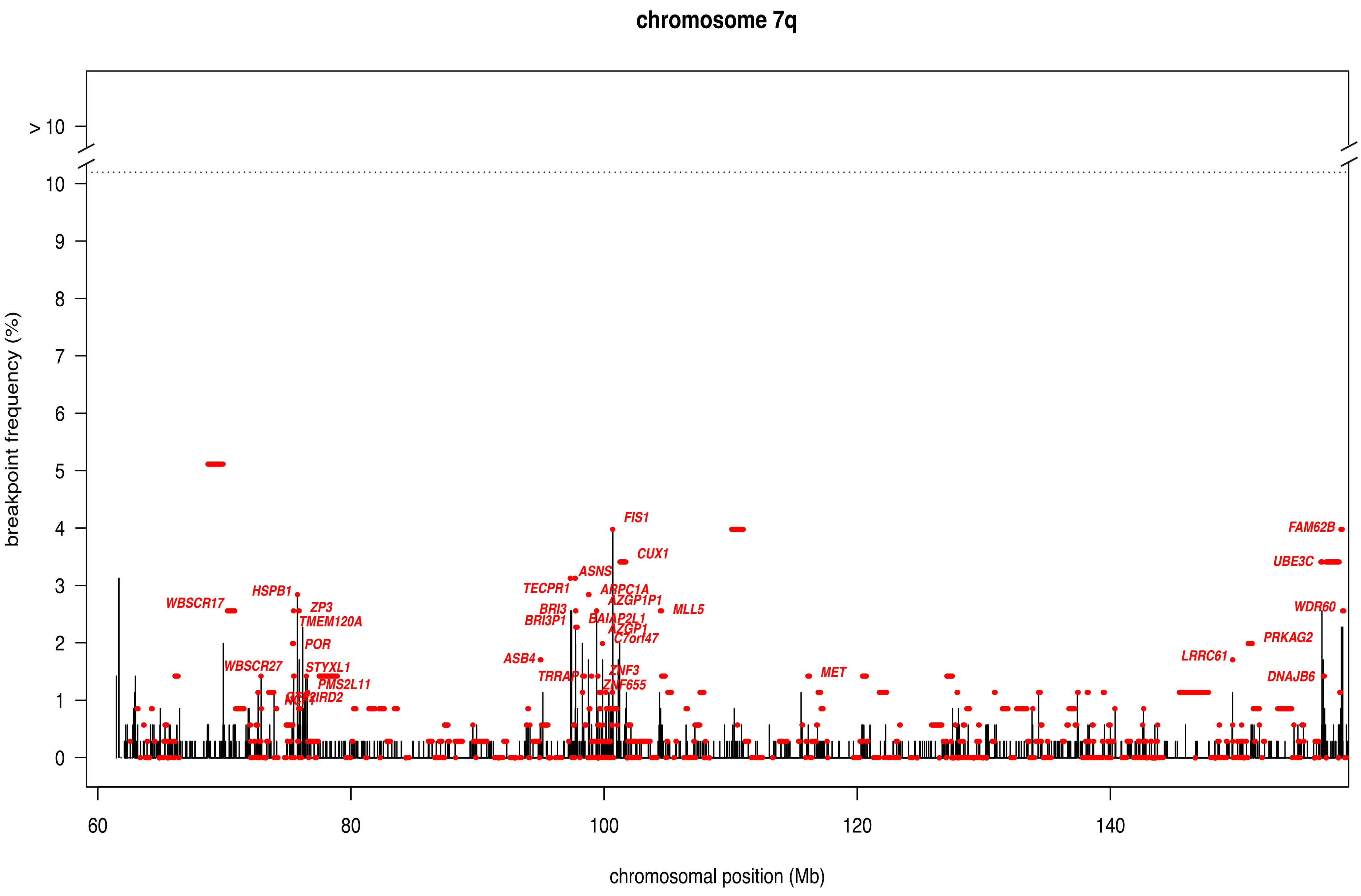

chromosome 7q
breakpoint frequency (%)
chromosomal position (Mb)
> 10
10
9
8
7
6
5
4
3
2
1
0
60
80
100
120
140
FIS1
FAM62B
CUX1
UBE3C
ASNS
TECPR1
ARPC1A
AZGP1P1
HSPB1
WBSCR17
ZP3
MLL5
BRI3
BRI3P1
BAIAP2L1
WDR60
TMEM120A
AZGP1
C7orf47
PRKAG2
POR
LRRC61
ASB4
WBSCR27
STYXL1
ZNF3
MET
TRRAP
DNAJB6
PMS2L11
ZNF655
GTF2IRD2
NCF4

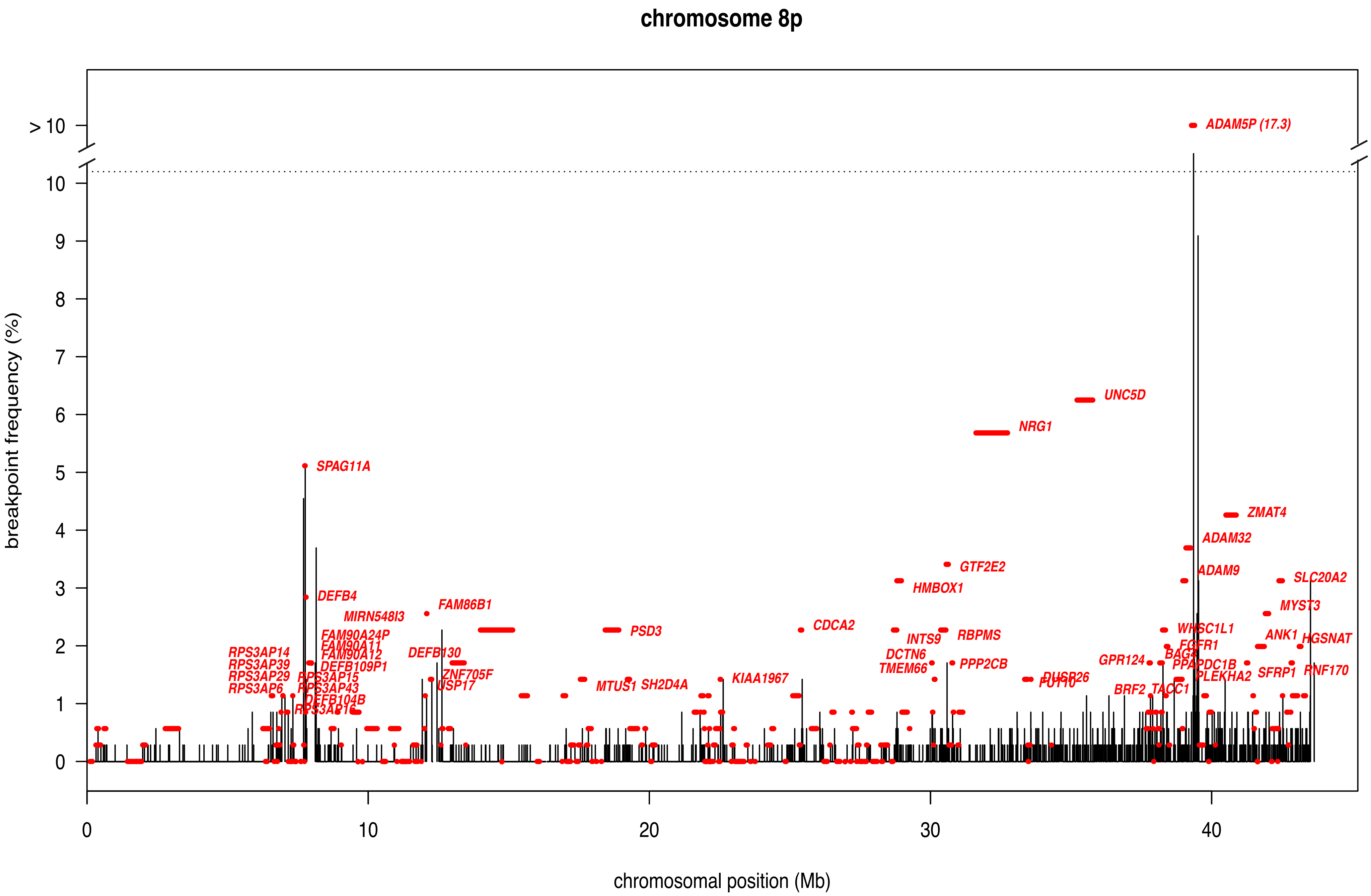
chromosome 8p
breakpoint frequency (%)
chromosomal position (Mb)
> 10
ADAM5P (17.3)
UNC5D
NRG1
SPAG11A
ZMAT4
ADAM32
GTF2E2
HMBOX1
ADAM9
SLC20A2
DEFB4
FAM86B1
MIRN548I3
MYST3
CDCA2
PSD3
WHSC1L1
INTS9
RBPMS
ANK1
HGSNAT
FAM90A24P
FAM90A11
FAM90A12
DEFB130
FGFR1
RPS3AP14
RPS3AP39
RPS3AP29
RPS3AP6
DCTN6
TMEM66
PPP2CB
GPR124
BAG4
PPAPDC1B
PLEKHA2
SFRP1
RNF170
DEFB109P1
RPS3AP15
RPS3AP43
ZNF705F
USP17
KIAA1967
DUSP26
FUT10
MTUS1
SH2D4A
BRF2
TACC1
DEFB104B
RPS3AP16

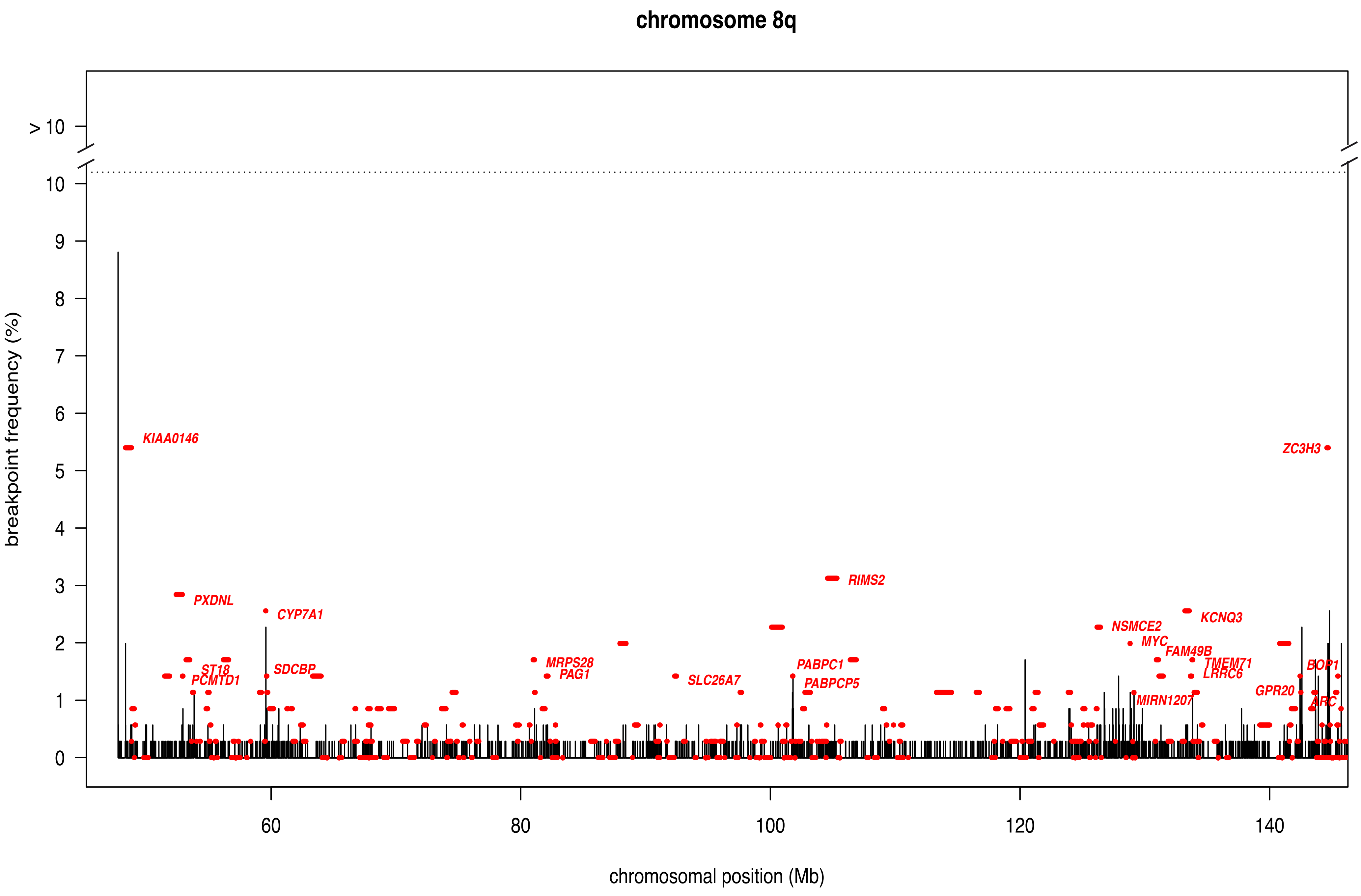
chromosome 8q
breakpoint frequency (%)
chromosomal position (Mb)
> 10
10
9
8
7
6
5
4
3
2
1
0
60
80
100
120
140
KIAA0146
ZC3H3
RIMS2
PXDNL
CYP7A1
KCNQ3
NSMCE2
MYC
FAM49B
ST18
PCMTD1
SDCBP
MRPS28
PAG1
SLC26A7
PABPC1
PABPCP5
TMEM71
LRRC6
BOP1
GPR20
MIRN1207
ARC

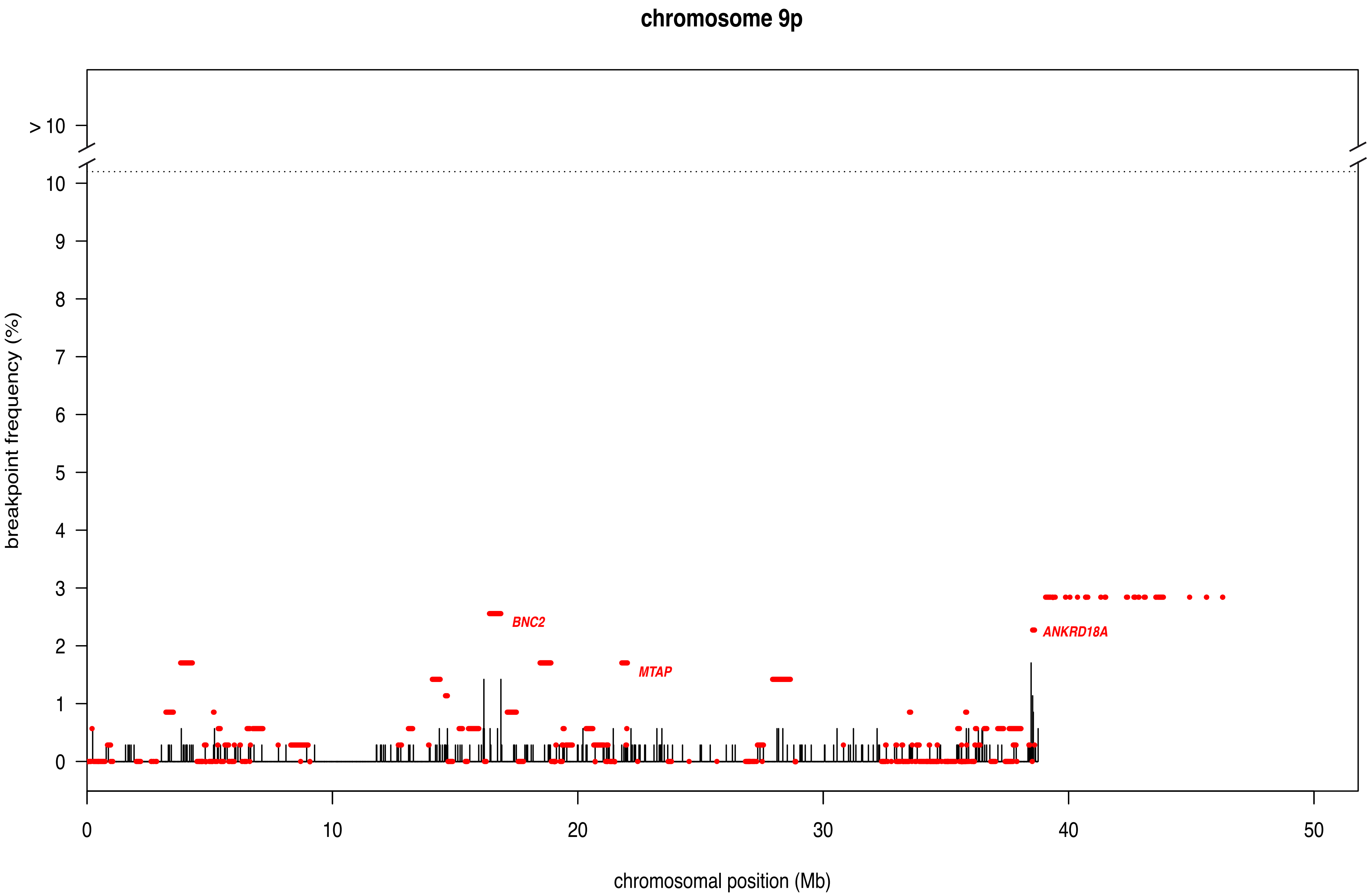
chromosome 9p
breakpoint frequency (%)
> 10
10
9
8
7
6
5
4
3
2
1
0
BNC2
MTAP
ANKRD18A
0
10
20
30
40
50
chromosomal position (Mb)

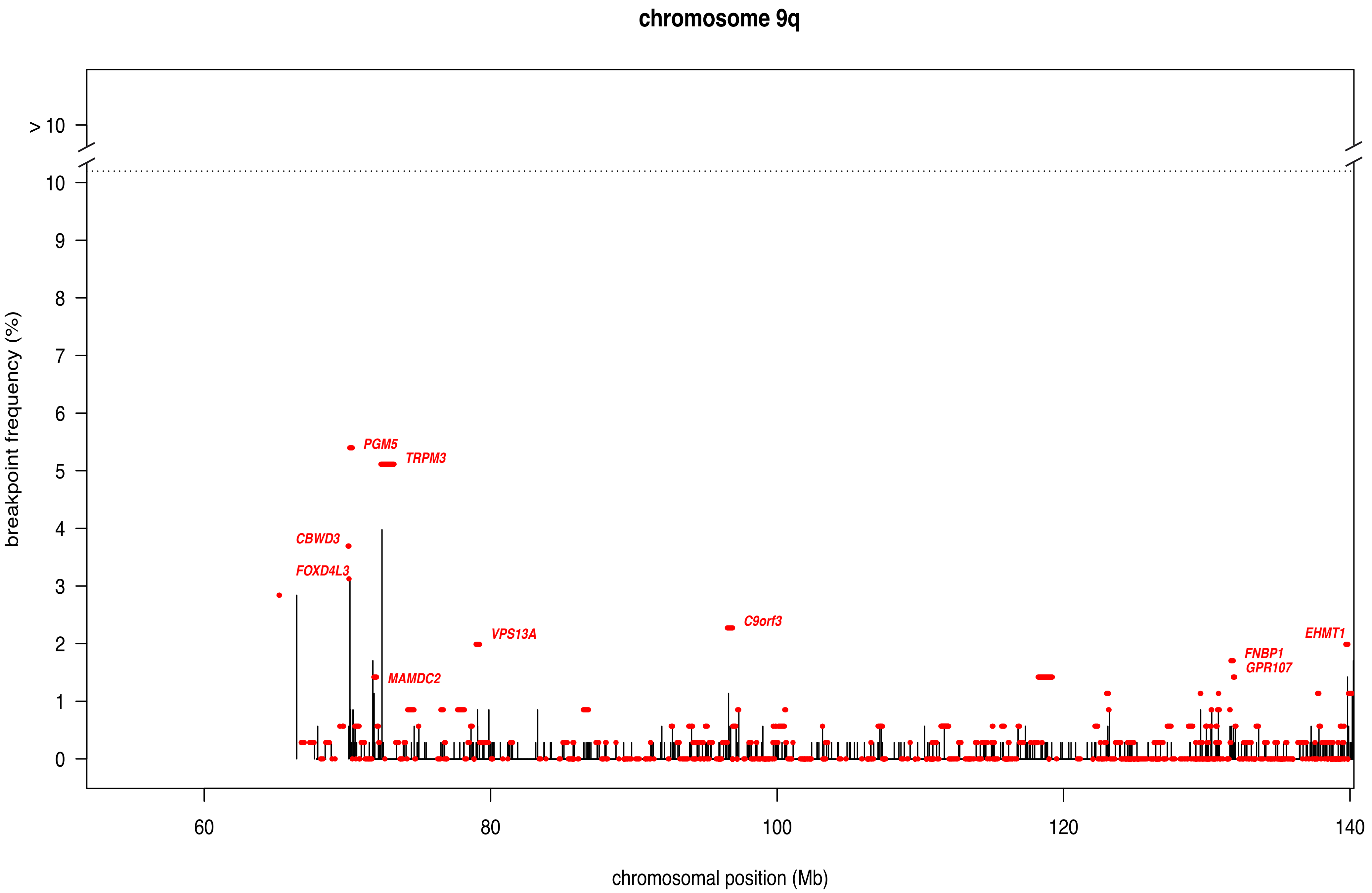

chromosome 9q
> 10
10
9
8
7
6
5
4
3
2
1
0
breakpoint frequency (%)
PGM5
TRPM3
CBWD3
FOXD4L3
VPS13A
C9orf3
MAMDC2
EHMT1
FNBP1
GPR107
60
80
100
120
140
chromosomal position (Mb)

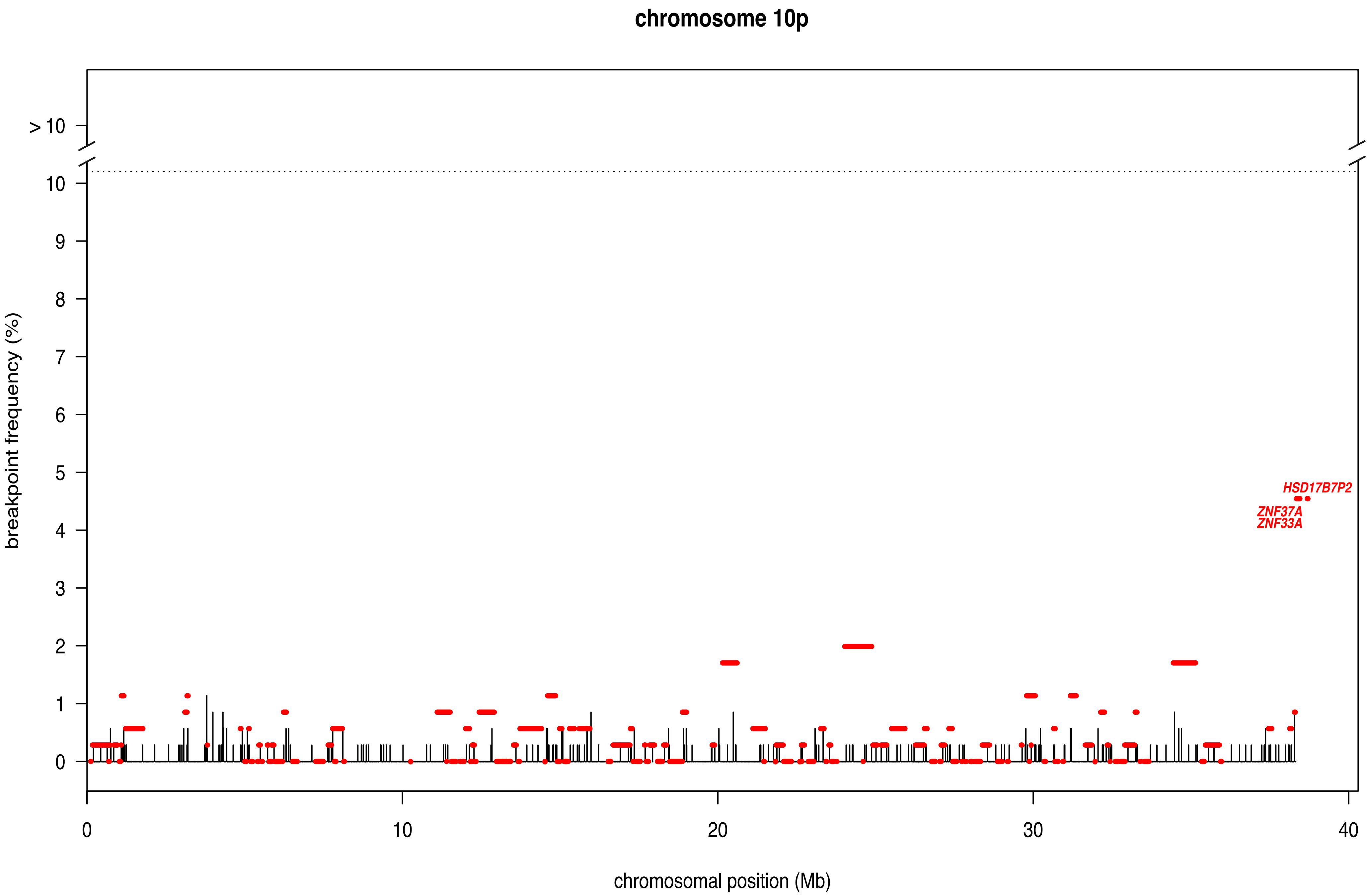
chromosome 10p
> 10
10
9
8
7
6
5
4
3
2
1
0
breakpoint frequency (%)
HSD17B7P2
ZNF37A
ZNF33A
0
10
20
30
40
chromosomal position (Mb)

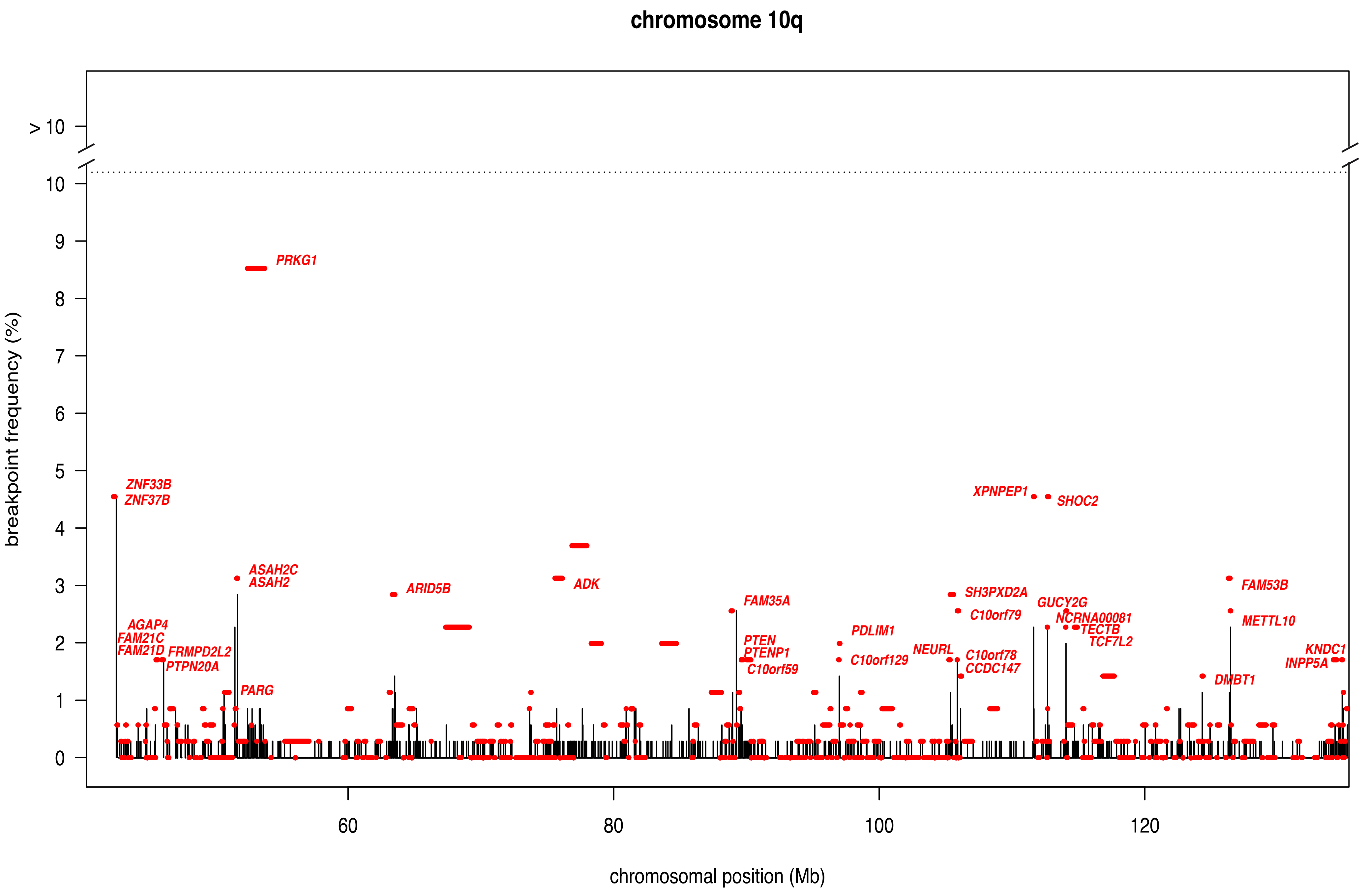

chromosome 10q
breakpoint frequency (%)
chromosomal position (Mb)
> 10
10
9
8
7
6
5
4
3
2
1
0
60
80
100
120
PRKG1
ZNF33B
ZNF37B
ASAH2C
ASAH2
ARID5B
ADK
AGAP4
FAM21C
FAM21D
FRMPD2L2
PTPN20A
PARG
FAM35A
PTEN
PTENP1
C10orf59
PDLIM1
C10orf129
NEURL
C10orf78
CCDC147
SH3PXD2A
C10orf79
XPNPEP1
SHOC2
GUCY2G
NCRNA00081
TECTB
TCF7L2
FAM53B
METTL10
DMBT1
KNDC1
INPP5A

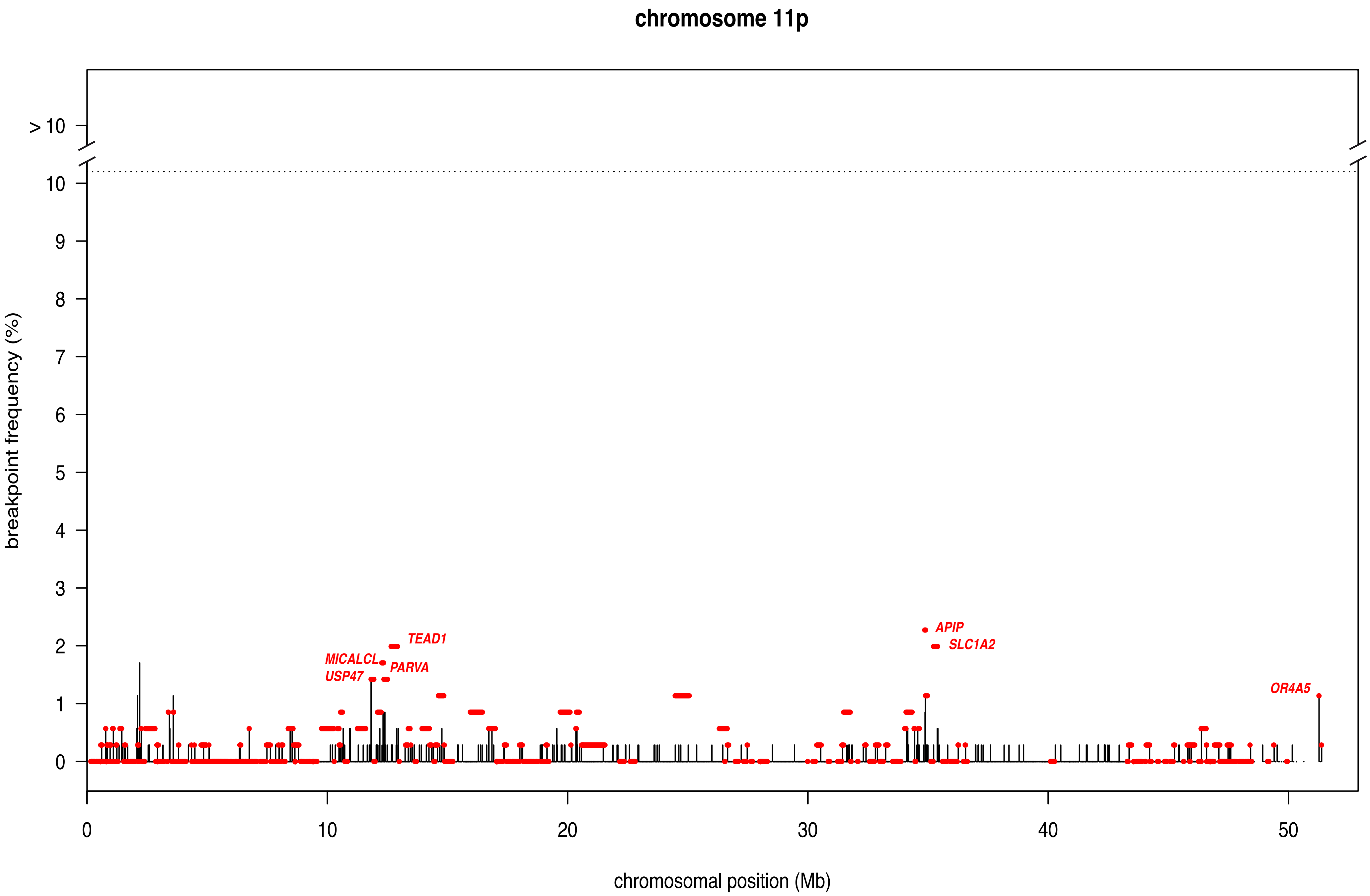

chromosome 11p
> 10
10
9
8
7
6
5
4
3
2
1
0
breakpoint frequency (%)
TEAD1
MICALCL
PARVA
USP47
APIP
SLC1A2
OR4A5
0
10
20
30
40
50
chromosomal position (Mb)

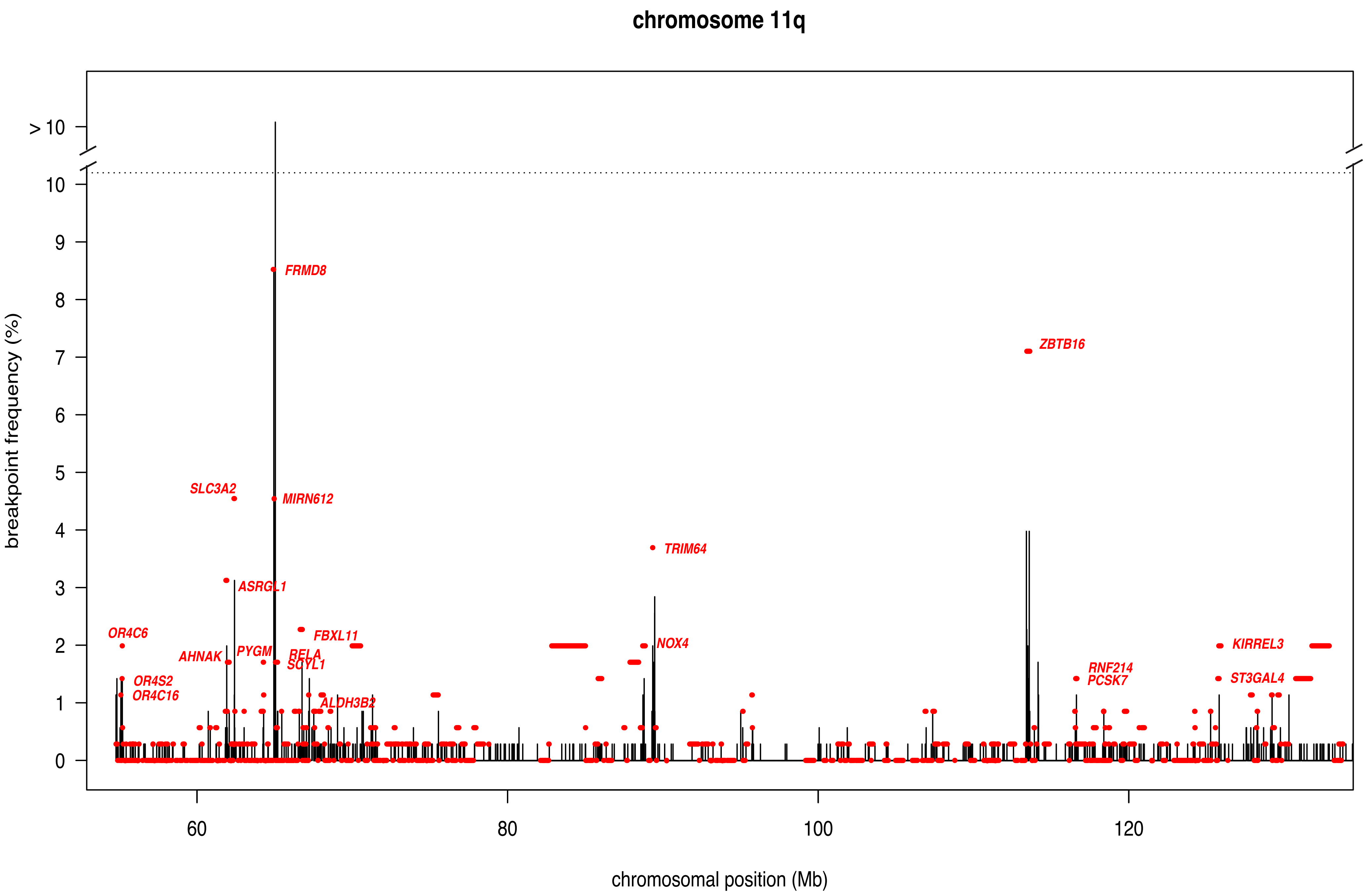

chromosome 11q
breakpoint frequency (%)
chromosomal position (Mb)
> 10
10
9
8
7
6
5
4
3
2
1
0
60
80
100
120
FRMD8
ZBTB16
SLC3A2
MIRN612
TRIM64
ASRGL1
OR4C6
FBXL11
NOX4
KIRREL3
AHNAK
PYGM
RELA
SCYL1
RNF214
PCSK7
ST3GAL4
OR4S2
OR4C16
ALDH3B2

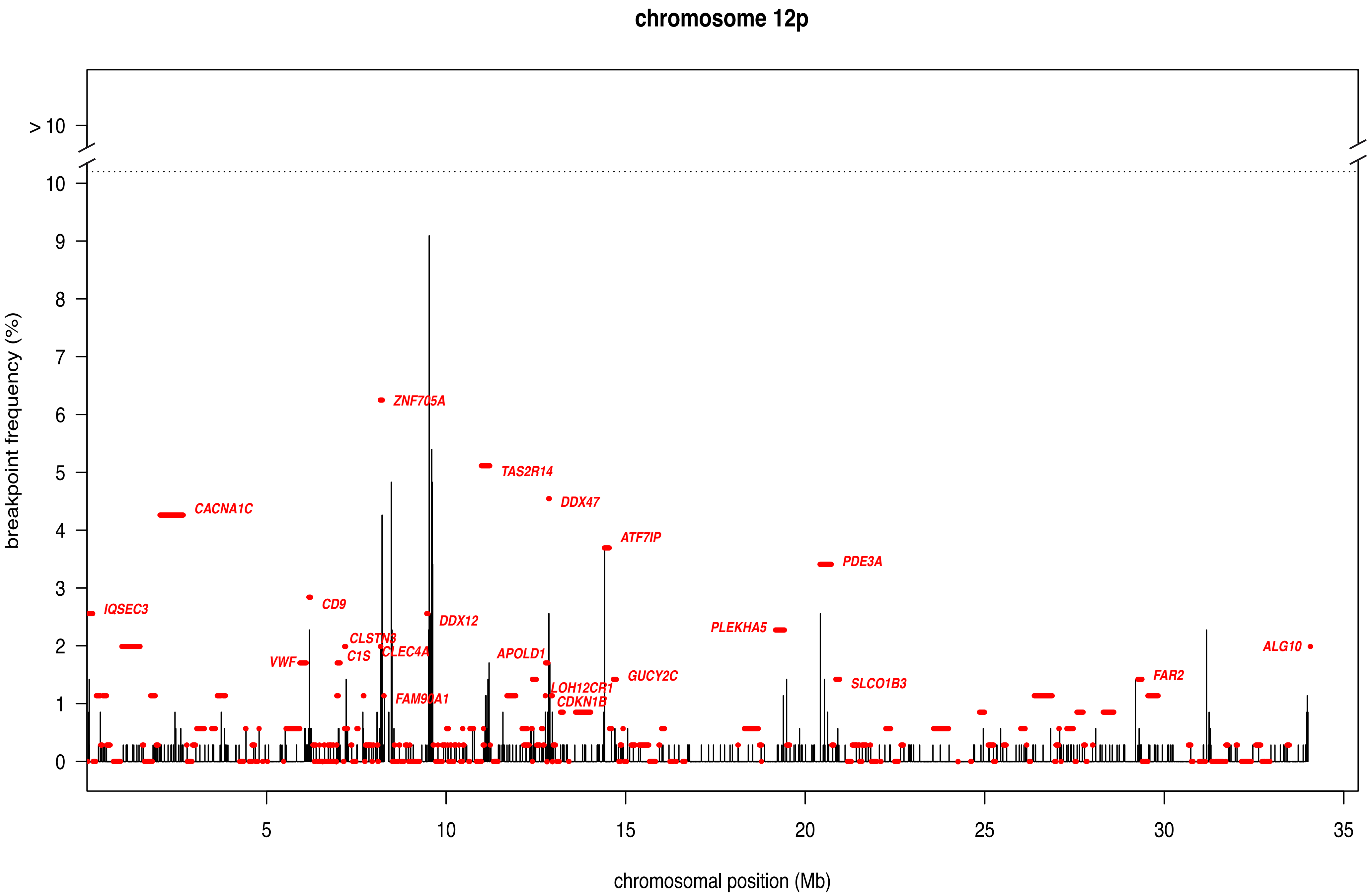
chromosome 12p
breakpoint frequency (%)
chromosomal position (Mb)
> 10
IQSEC3
CACNA1C
CD9
VWF
CLSTN3
C1S
CLEC4A
ZNF705A
FAM90A1
DDX12
TAS2R14
APOLD1
DDX47
LOH12CR1
CDKN1B
ATF7IP
GUCY2C
PLEKHA5
PDE3A
SLCO1B3
FAR2
ALG10

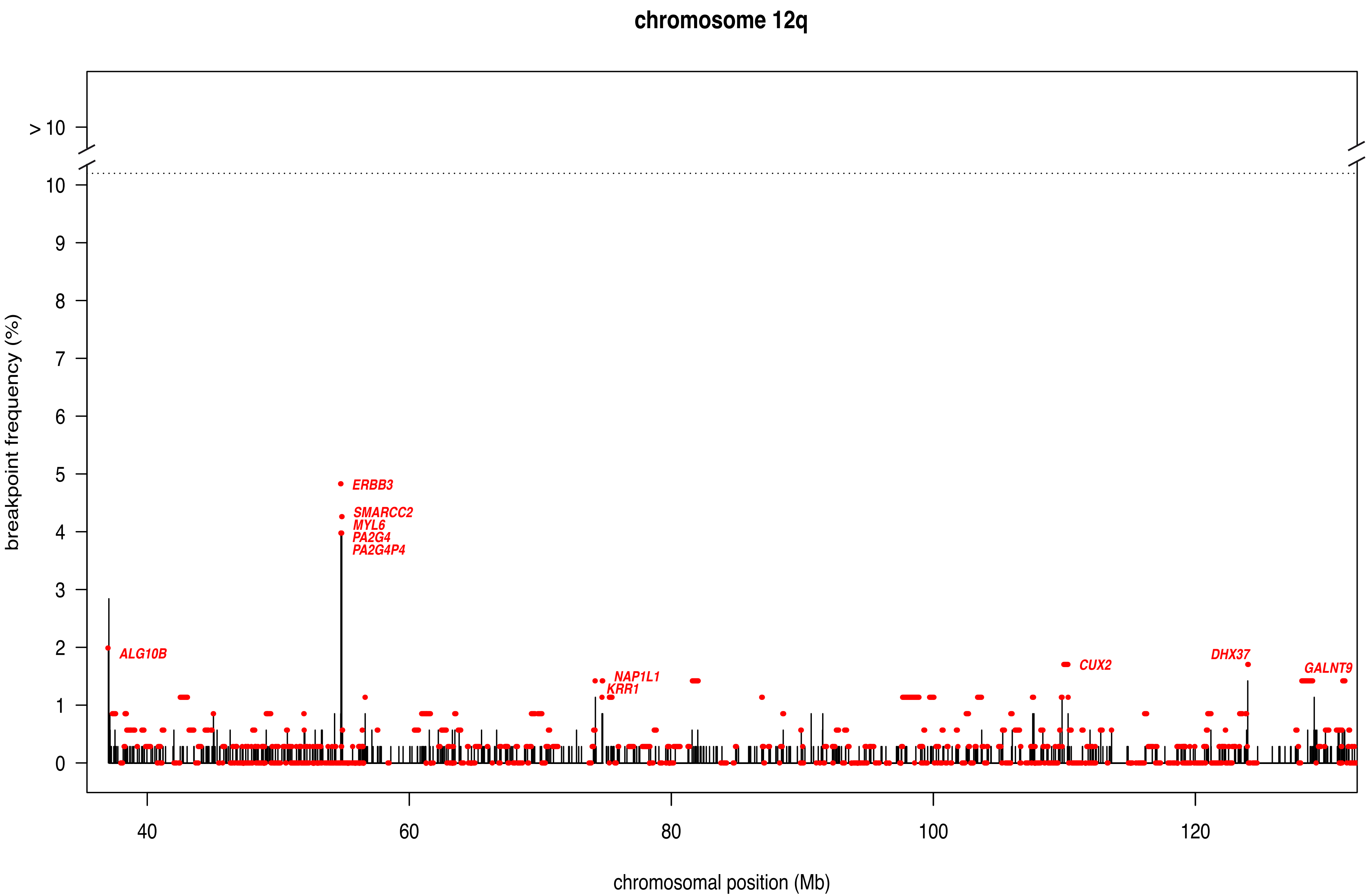

chromosome 12q
breakpoint frequency (%)
chromosomal position (Mb)
> 10
10
9
8
7
6
5
4
3
2
1
0
40
60
80
100
120
ERBB3
SMARCC2
MYL6
PA2G4
PA2G4P4
ALG10B
NAP1L1
KRR1
CUX2
DHX37
GALNT9

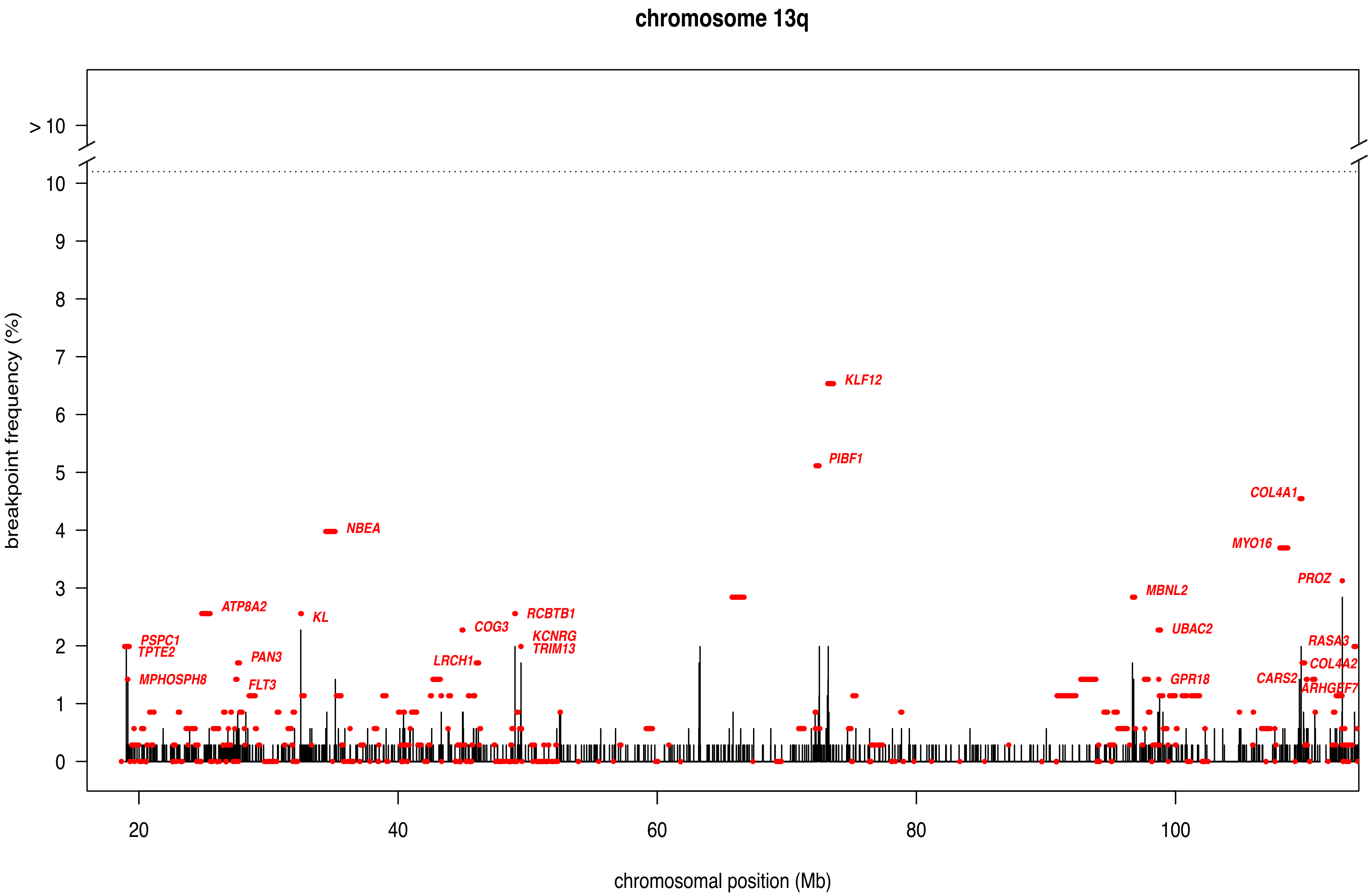
chromosome 13q
breakpoint frequency (%)
chromosomal position (Mb)
> 10
10
9
8
7
6
5
4
3
2
1
0
20
40
60
80
100
KLF12
PIBF1
COL4A1
NBEA
MYO16
PROZ
MBNL2
ATP8A2
KL
RCBTB1
COG3
UBAC2
KCNRG
TRIM13
PSPC1
TPTE2
RASA3
PAN3
LRCH1
COL4A2
MPHOSPH8
GPR18
CARS2
FLT3
ARHGEF7

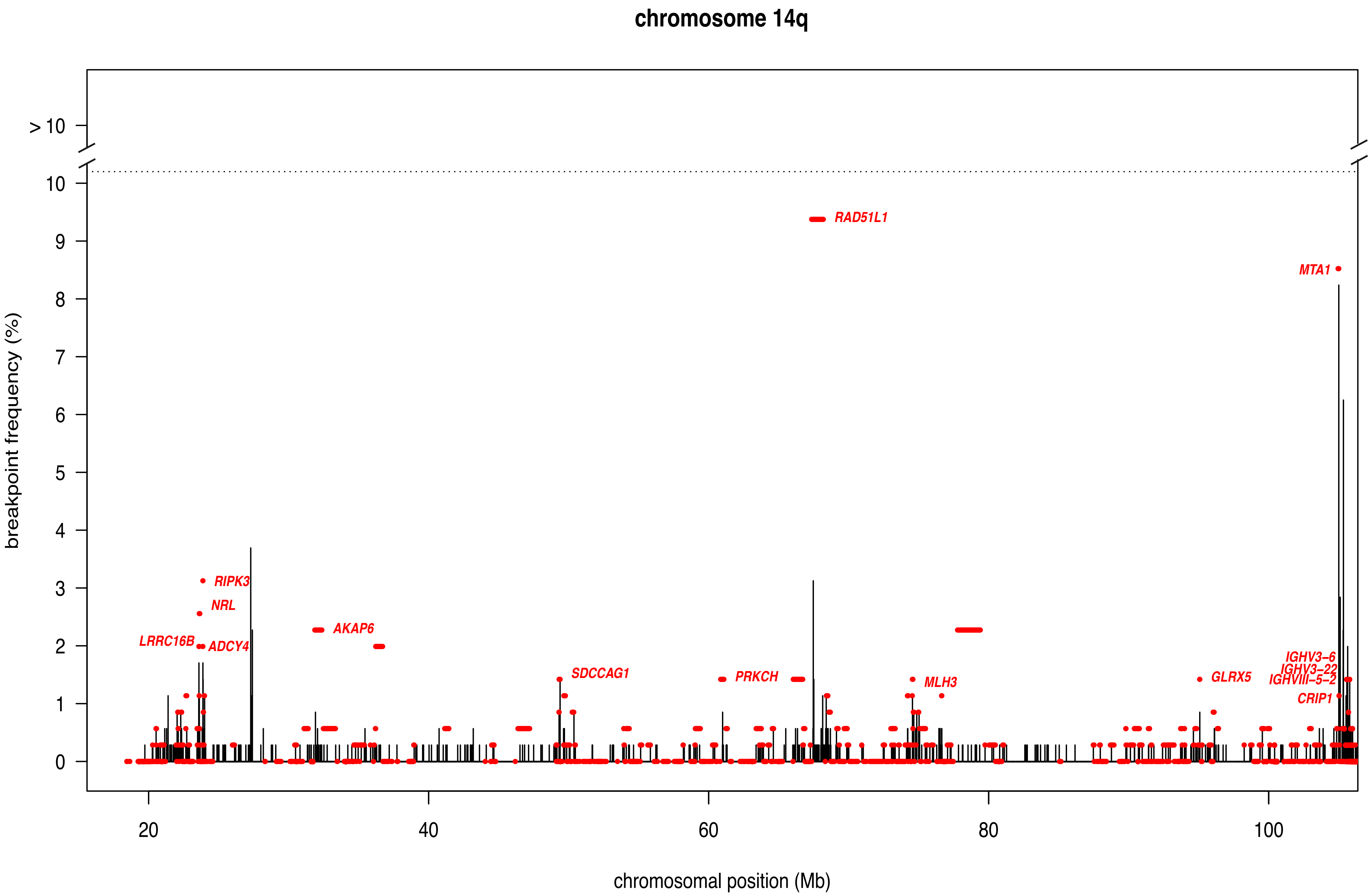

chromosome 14q
breakpoint frequency (%)
chromosomal position (Mb)
> 10
10
9
8
7
6
5
4
3
2
1
0
20
40
60
80
100
RAD51L1
MTA1
RIPK3
NRL
LRRC16B
ADCY4
AKAP6
SDCCAG1
PRKCH
MLH3
GLRX5
IGHV3-6
IGHV3-22
IGHVII-5-2
CRIP1

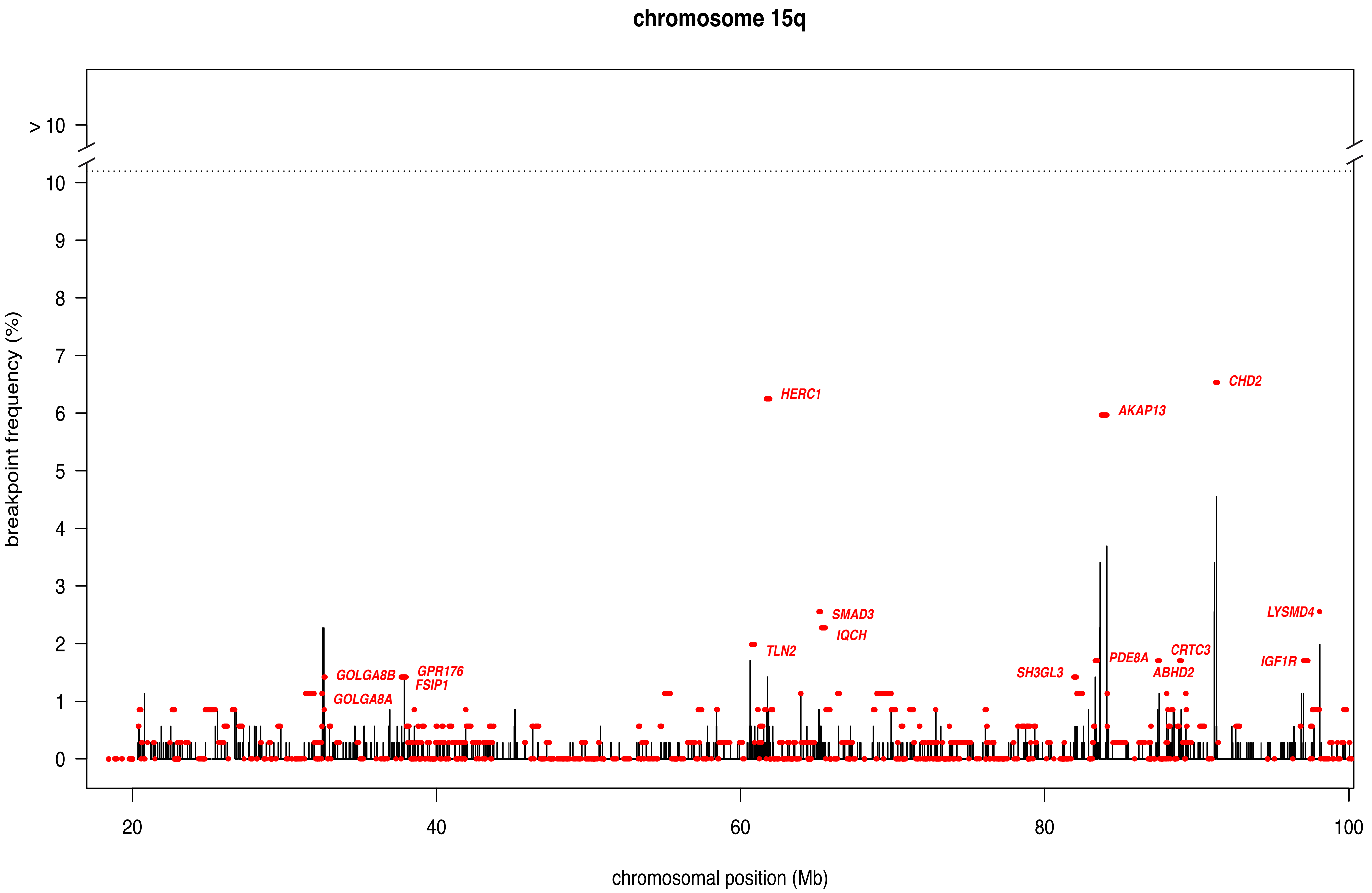
chromosome 15q
> 10
10
9
8
7
6
5
4
3
2
1
0
breakpoint frequency (%)
HERC1
CHD2
AKAP13
SMAD3
IQCH
LYSMD4
TLN2
CRTC3
PDE8A
ABHD2
SH3GL3
IGF1R
GOLGA8B
GPR176
FSIP1
GOLGA8A
20
40
60
80
100
chromosomal position (Mb)

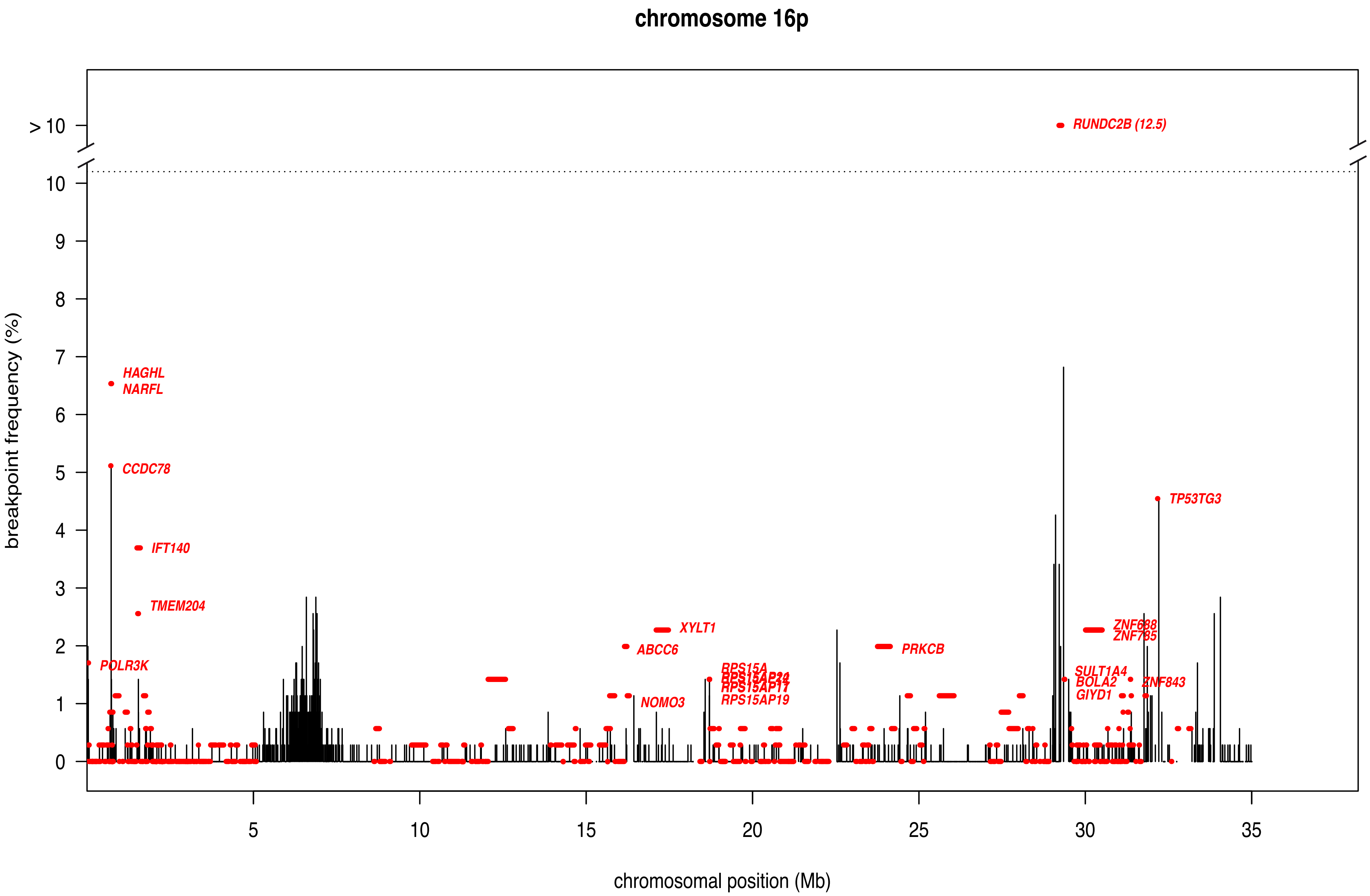

chromosome 16p
breakpoint frequency (%)
chromosomal position (Mb)
> 10
RUNDC2B (12.5)
HAGHL
NARFL
CCDC78
IFT140
TMEM204
POLR3K
XYLT1
ABCC6
NOMO3
RPS15A
RPS15AP17
RPS15AP19
PRKCB
ZNF688
ZNF785
SULT1A4
BOLA2
GIYD1
ZNF843
TP53TG3

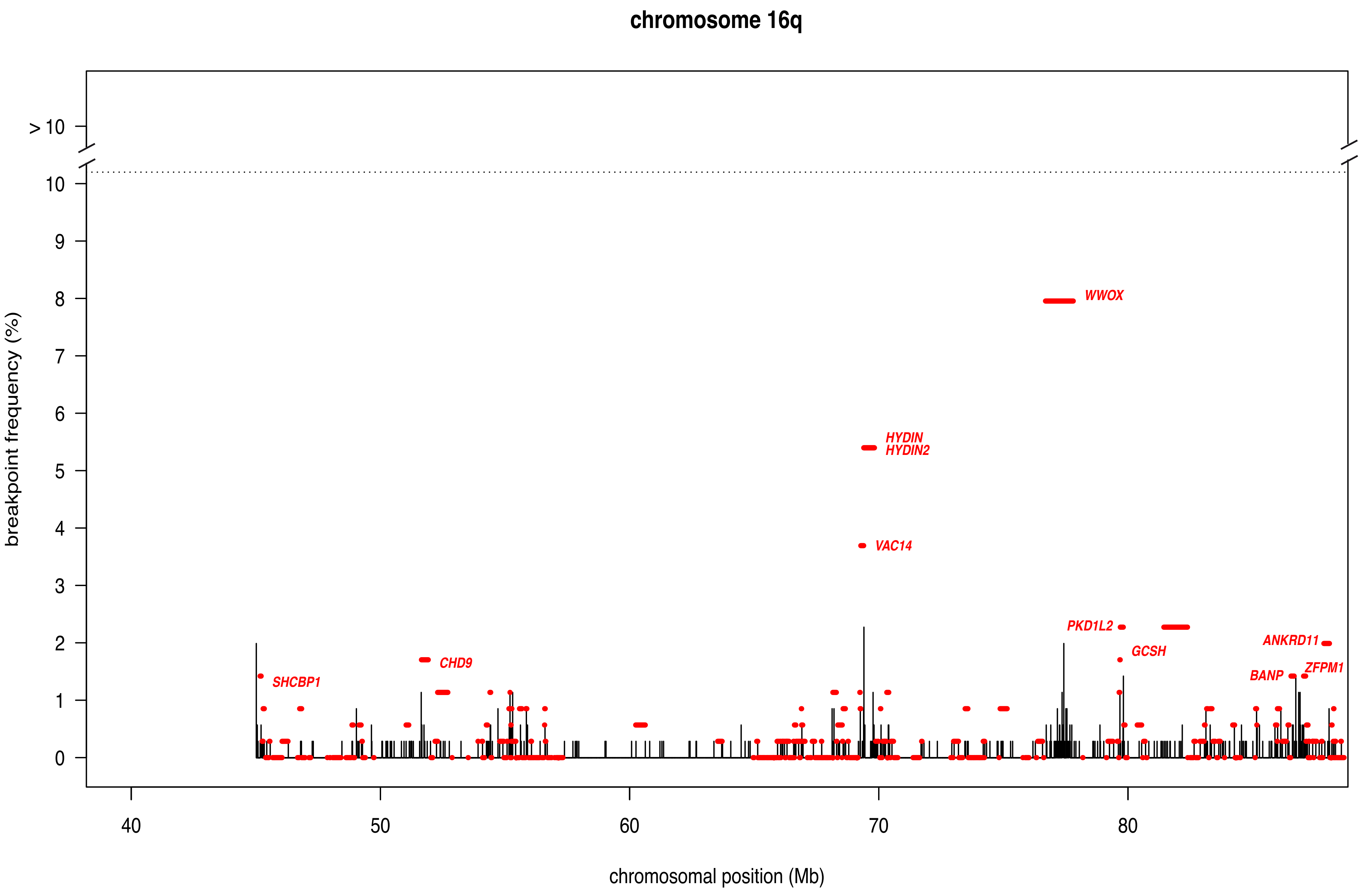

chromosome 16q
breakpoint frequency (%)
chromosomal position (Mb)
> 10
10
9
8
7
6
5
4
3
2
1
0
40
50
60
70
80
WWOX
HYDIN
HYDIN2
VAC14
PKD1L2
GCSH
ANKRD11
BANP
ZFPM1
CHD9
SHCBP1

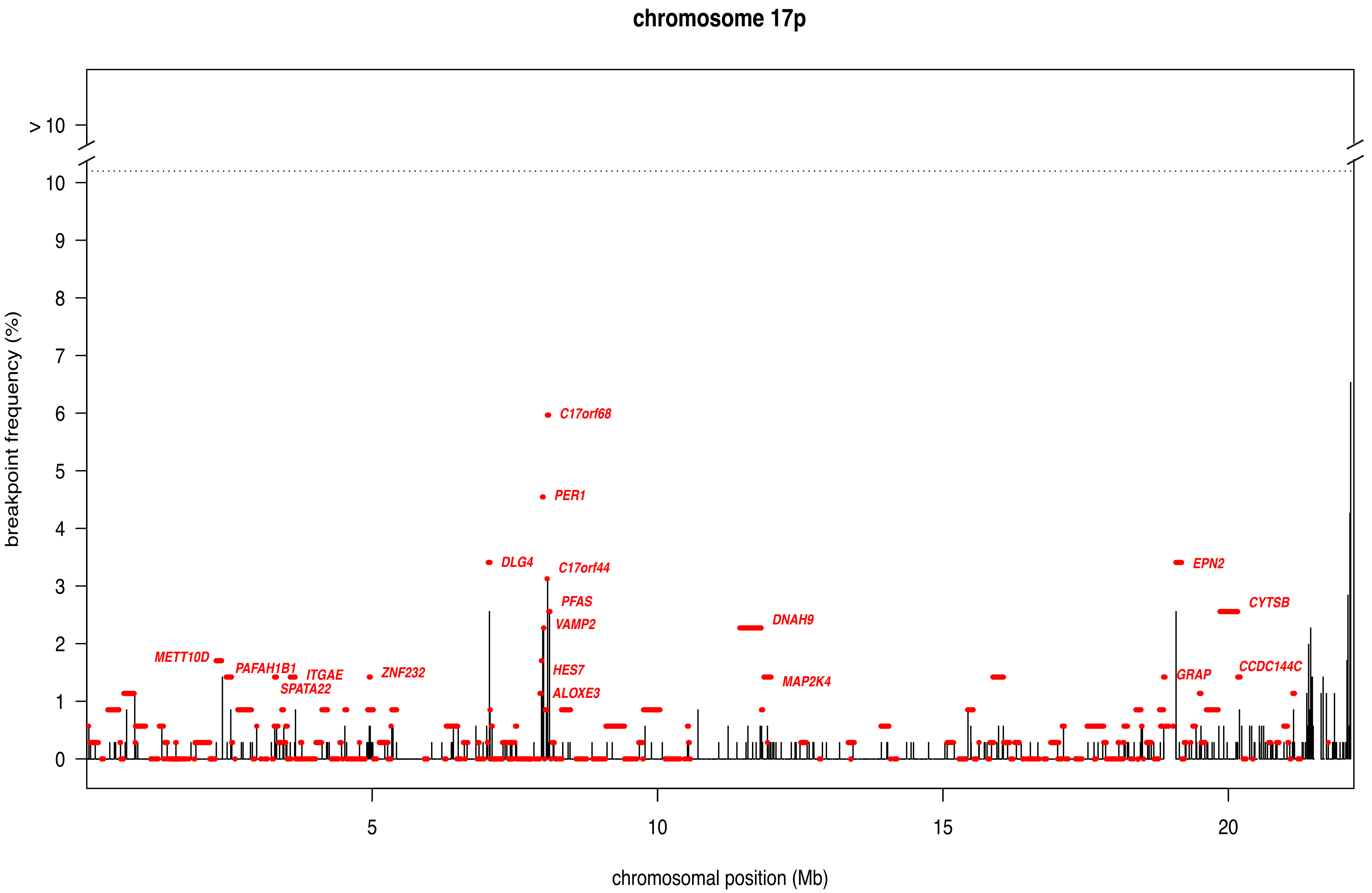
chromosome 17p
breakpoint frequency (%)
chromosomal position (Mb)
> 10
10
9
8
7
6
5
4
3
2
1
0
5
10
15
20
C17orf68
PER1
DLG4
C17orf44
PFAS
VAMP2
HES7
ALOXE3
METT10D
PAFAH1B1
ITGAE
SPATA22
ZNF232
DNAH9
MAP2K4
EPN2
CYTSB
GRAP
CCDC144C

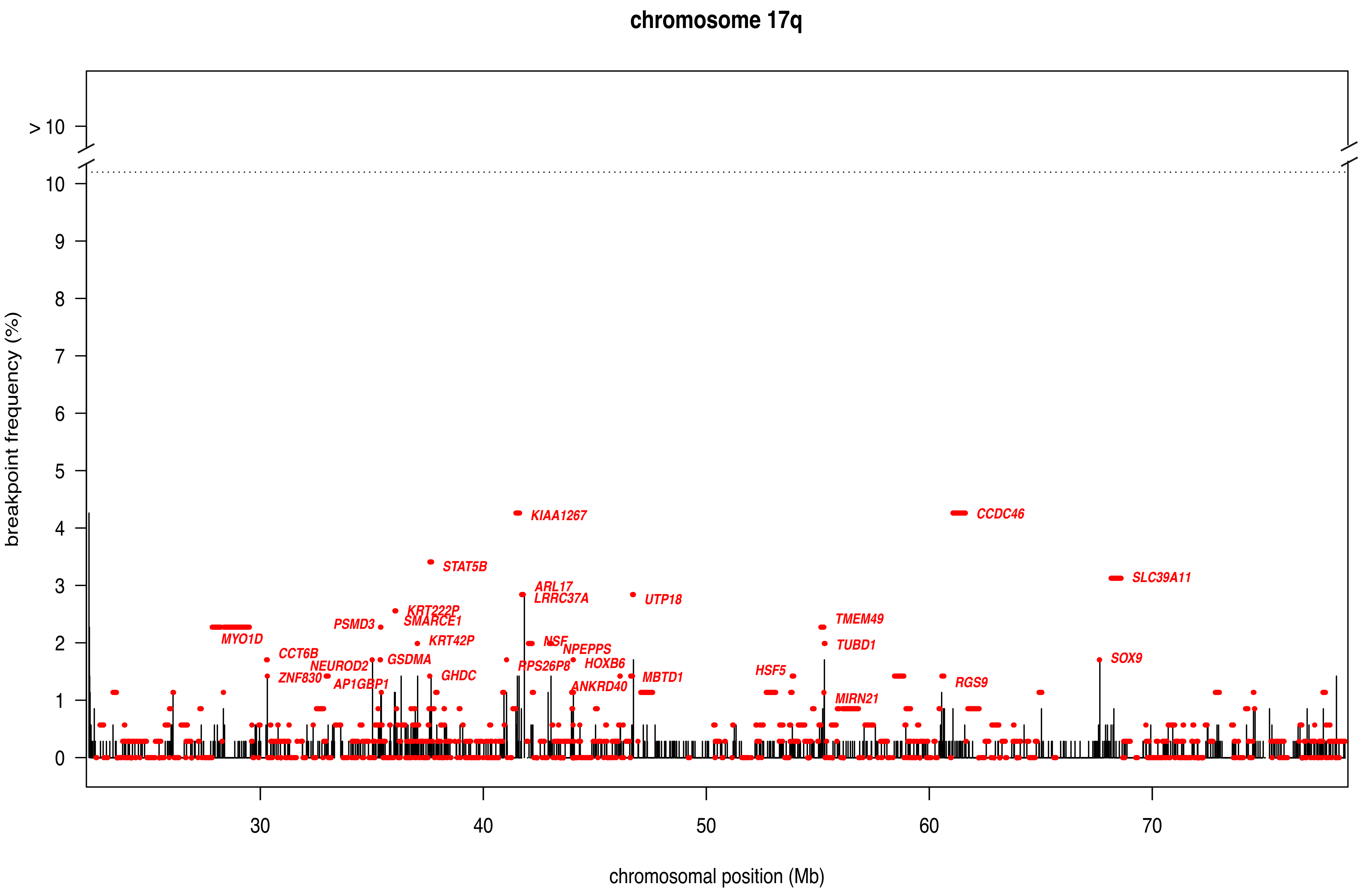

chromosome 17q
breakpoint frequency (%)
chromosomal position (Mb)
> 10
10
9
8
7
6
5
4
3
2
1
0
30
40
50
60
70
KIAA1267
CCDC46
STAT5B
SLC39A11
ARL17
LRRC37A
UTP18
KRT222P
SMARCE1
PSMD3
TMEM49
MYO1D
KRT42P
NSF
NPEPPS
TUBD1
CCT6B
GSDMA
NEUROD2
RPS26P8
HOXB6
SOX9
HSF5
GHDC
ZNF830
MBTD1
AP1GBP1
ANKRD40
RGS9
MIRN21

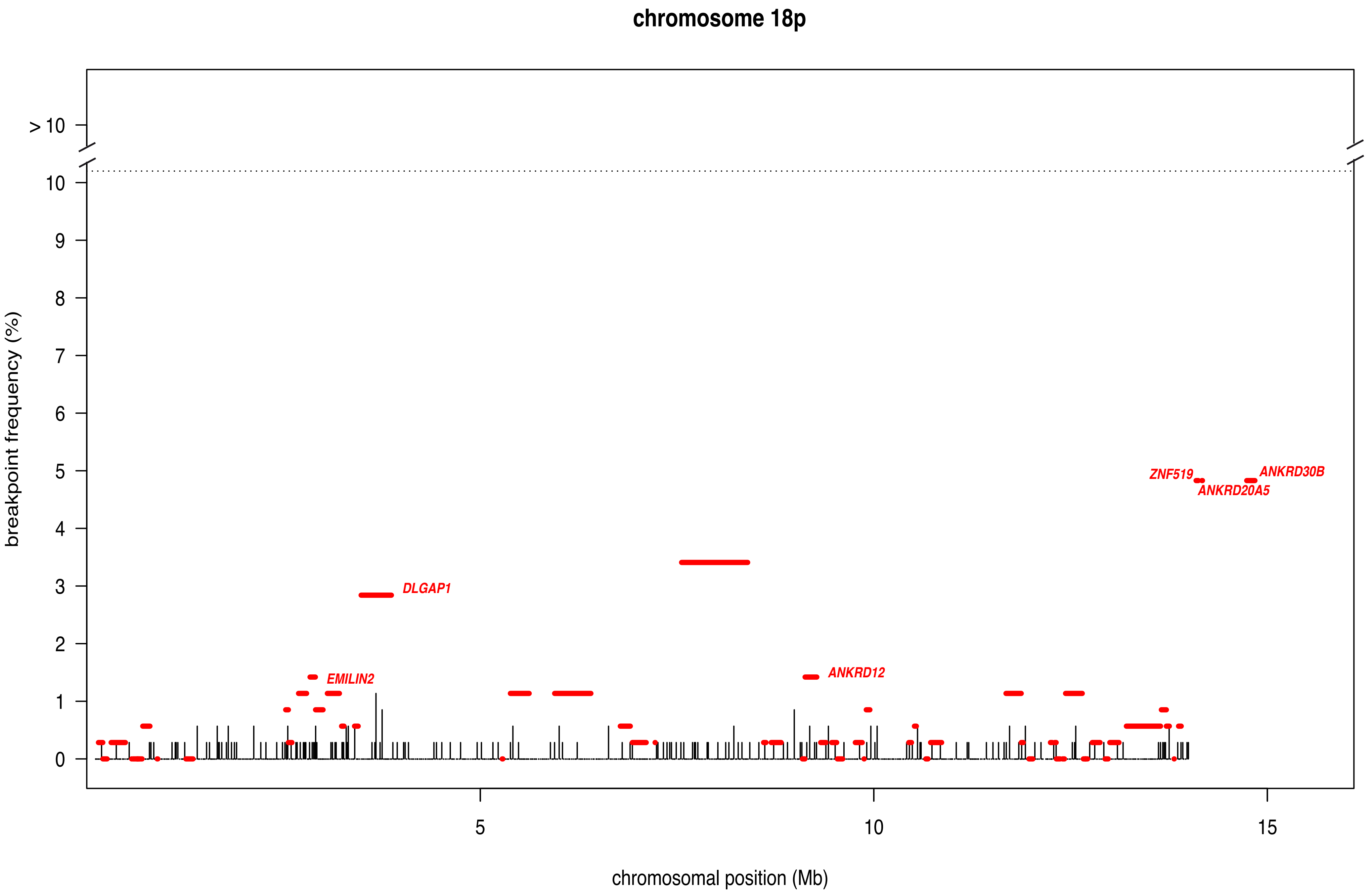
chromosome 18p
breakpoint frequency (%)
> 10
10
9
8
7
6
5
4
3
2
1
0
DLGAP1
EMILIN2
ANKRD12
ZNF519
ANKRD30B
ANKRD20A5
5
10
15
chromosomal position (Mb)

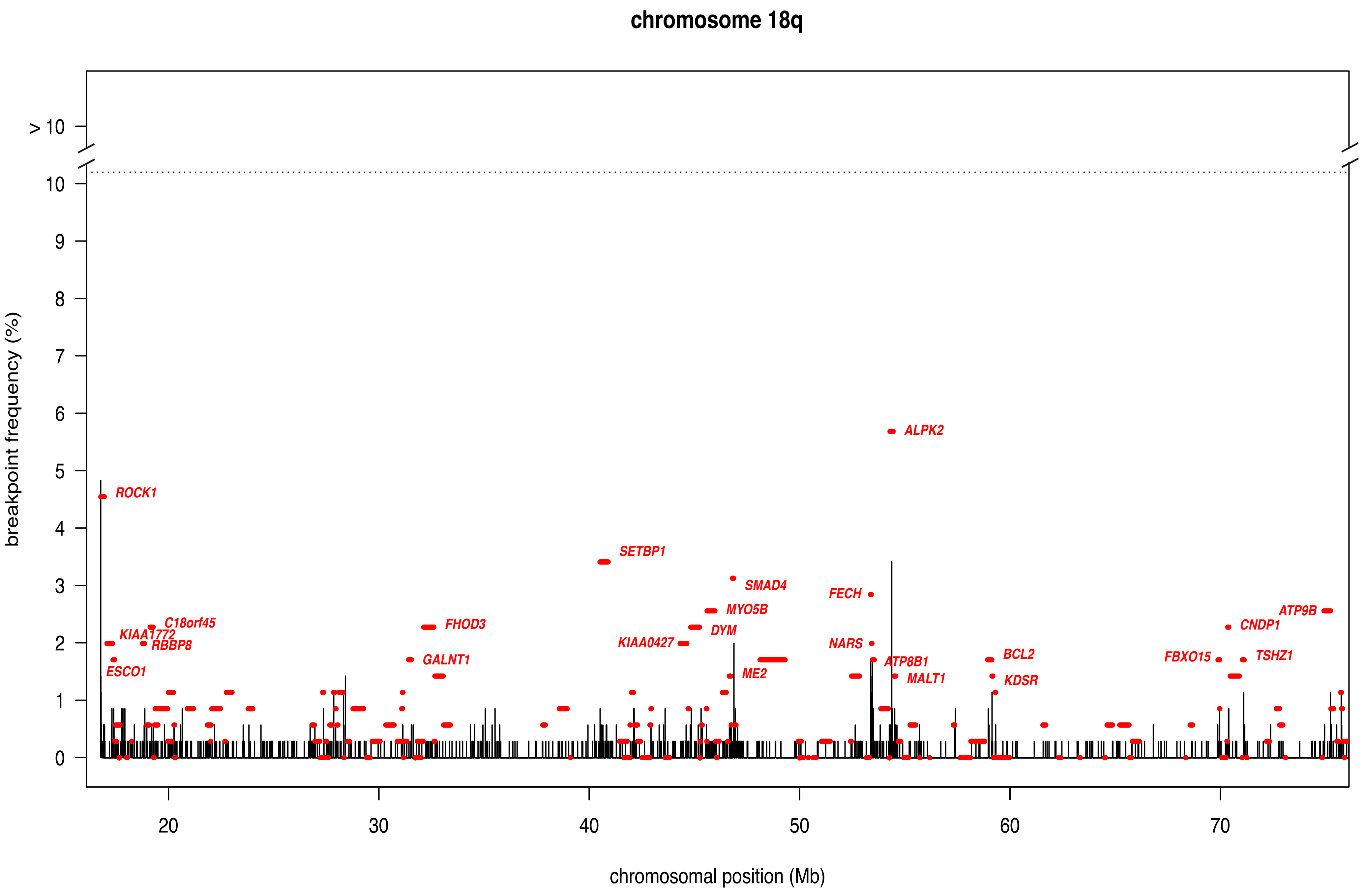

chromosome 18q
breakpoint frequency (%)
chromosomal position (Mb)
> 10
10
9
8
7
6
5
4
3
2
1
0
20
30
40
50
60
70
ROCK1
KIAA1772
C18orf45
RBBP8
ESCO1
FHOD3
GALNT1
SETBP1
KIAA0427
DYM
MYO5B
SMAD4
ME2
FECH
NARS
ATP8B1
MALT1
ALPK2
BCL2
KDSR
FBXO15
CNDP1
TSHZ1
ATP9B

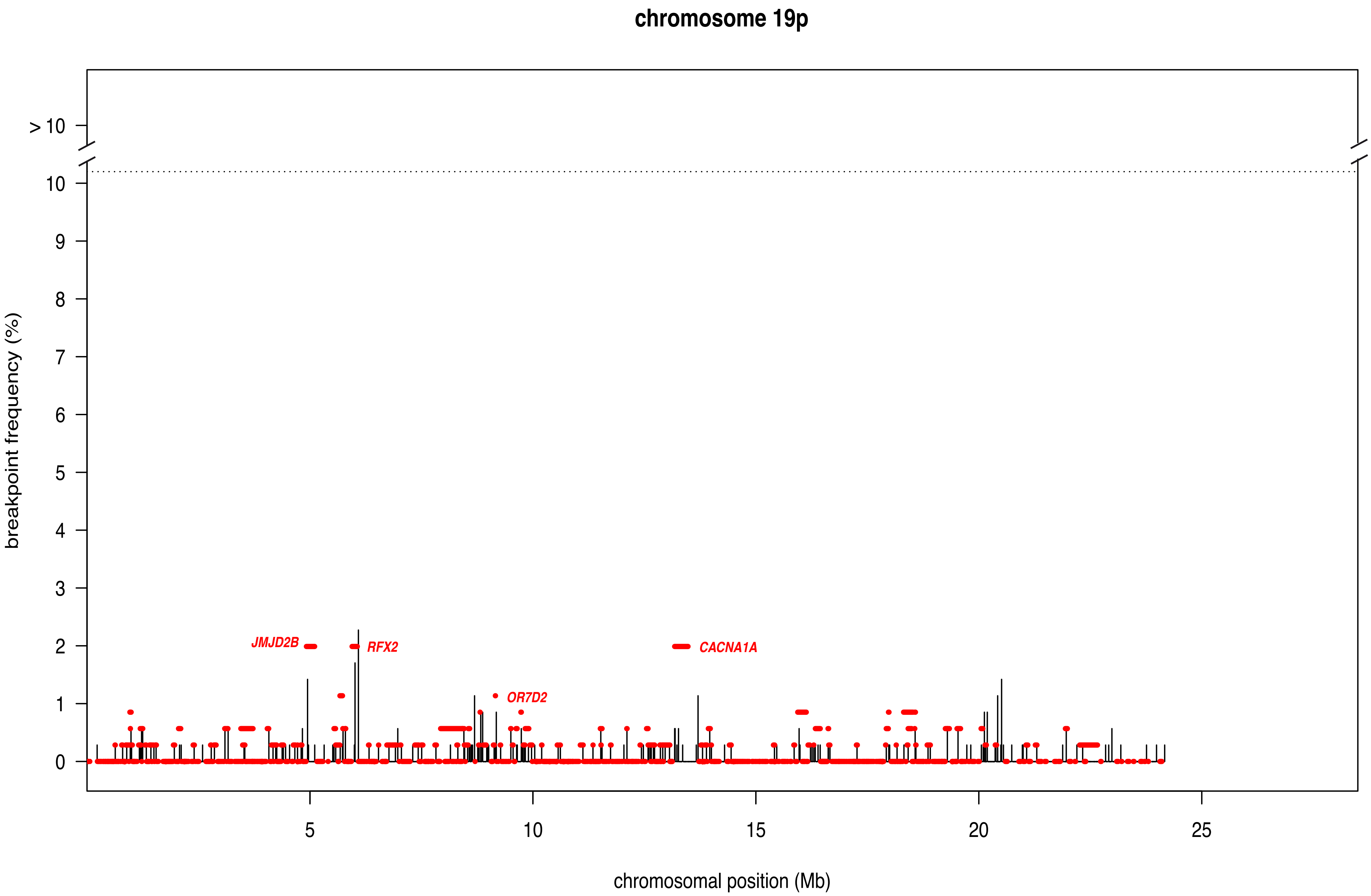
chromosome 19p
> 10
10
9
8
7
6
5
4
3
2
1
0
breakpoint frequency (%)
JMJD2B
RFX2
OR7D2
CACNA1A
5
10
15
20
25
chromosomal position (Mb)

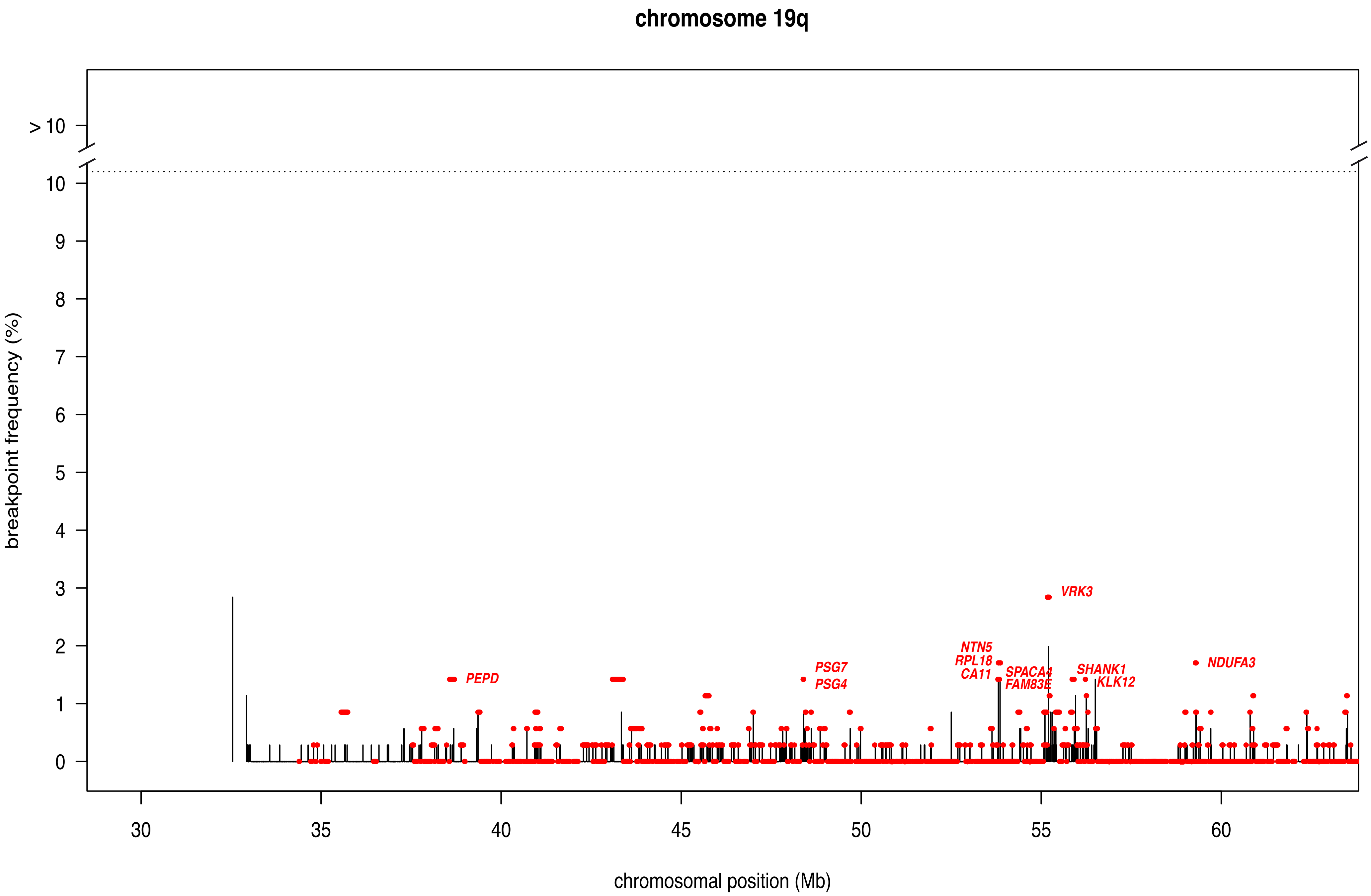
chromosome 19q
breakpoint frequency (%)
chromosomal position (Mb)
> 10
0
1
2
3
4
5
6
7
8
9
10
30
35
40
45
50
55
60
PEPD
PSG7
PSG4
NTN5
RPL18
CA11
SPACA4
FAM83E
VRK3
SHANK1
KLK12
NDUFA3

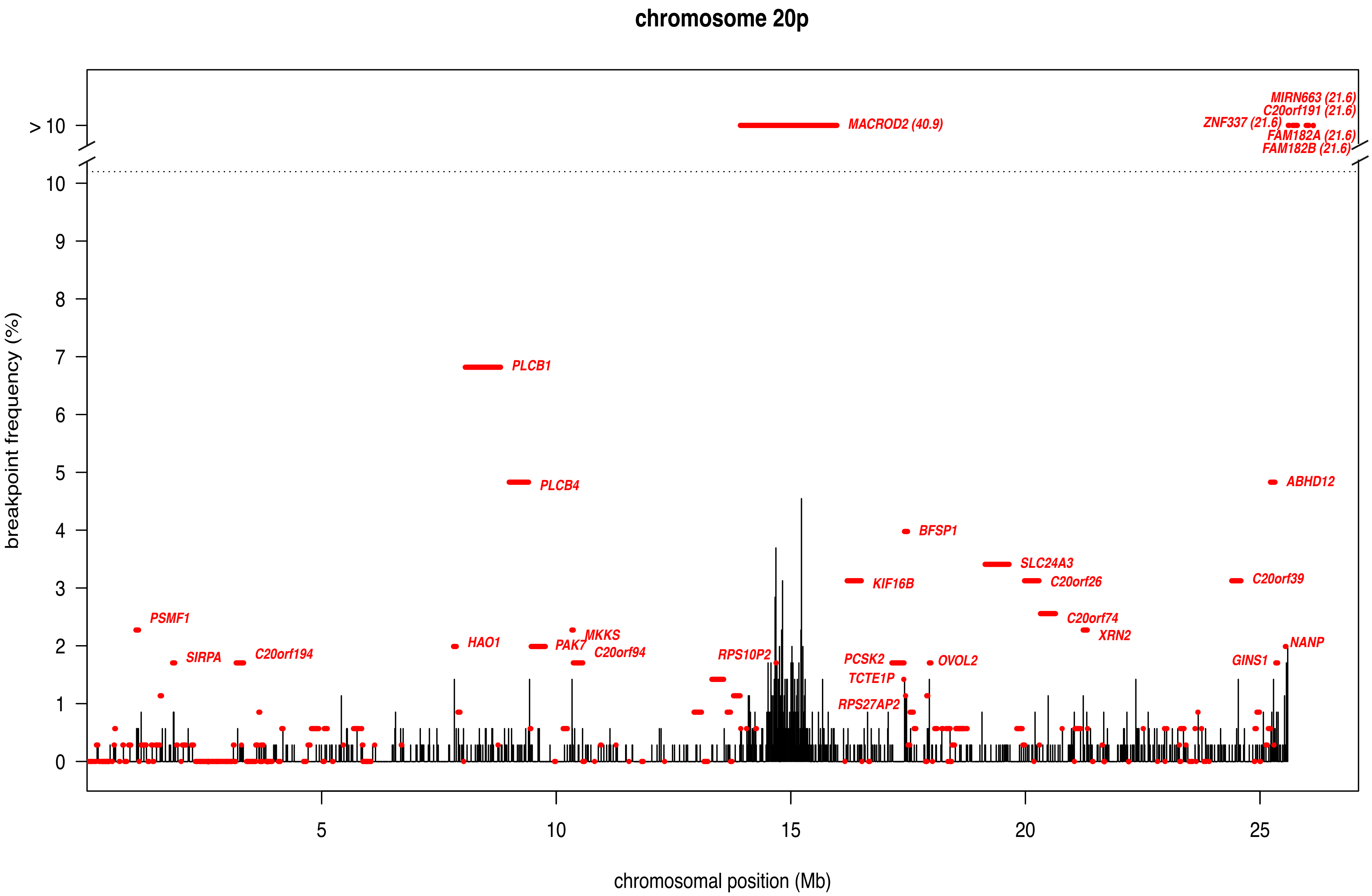

chromosome 20p
breakpoint frequency (%)
chromosomal position (Mb)
> 10
0
1
2
3
4
5
6
7
8
9
10
5
10
15
20
25
MACROD2 (40.9)
MIRN663 (21.6)
C20orf191 (21.6)
ZNF337 (21.6)
FAM182A (21.6)
FAM182B (21.6)
PLCB1
PLCB4
ABHD12
BFSP1
SLC24A3
KIF16B
C20orf26
C20orf39
C20orf74
XRN2
PSMF1
MKKS
HAO1
PAK7
C20orf94
SIRPA
C20orf194
RPS10P2
PCSK2
OVOL2
NANP
GINS1
TCTE1P
RPS27AP2

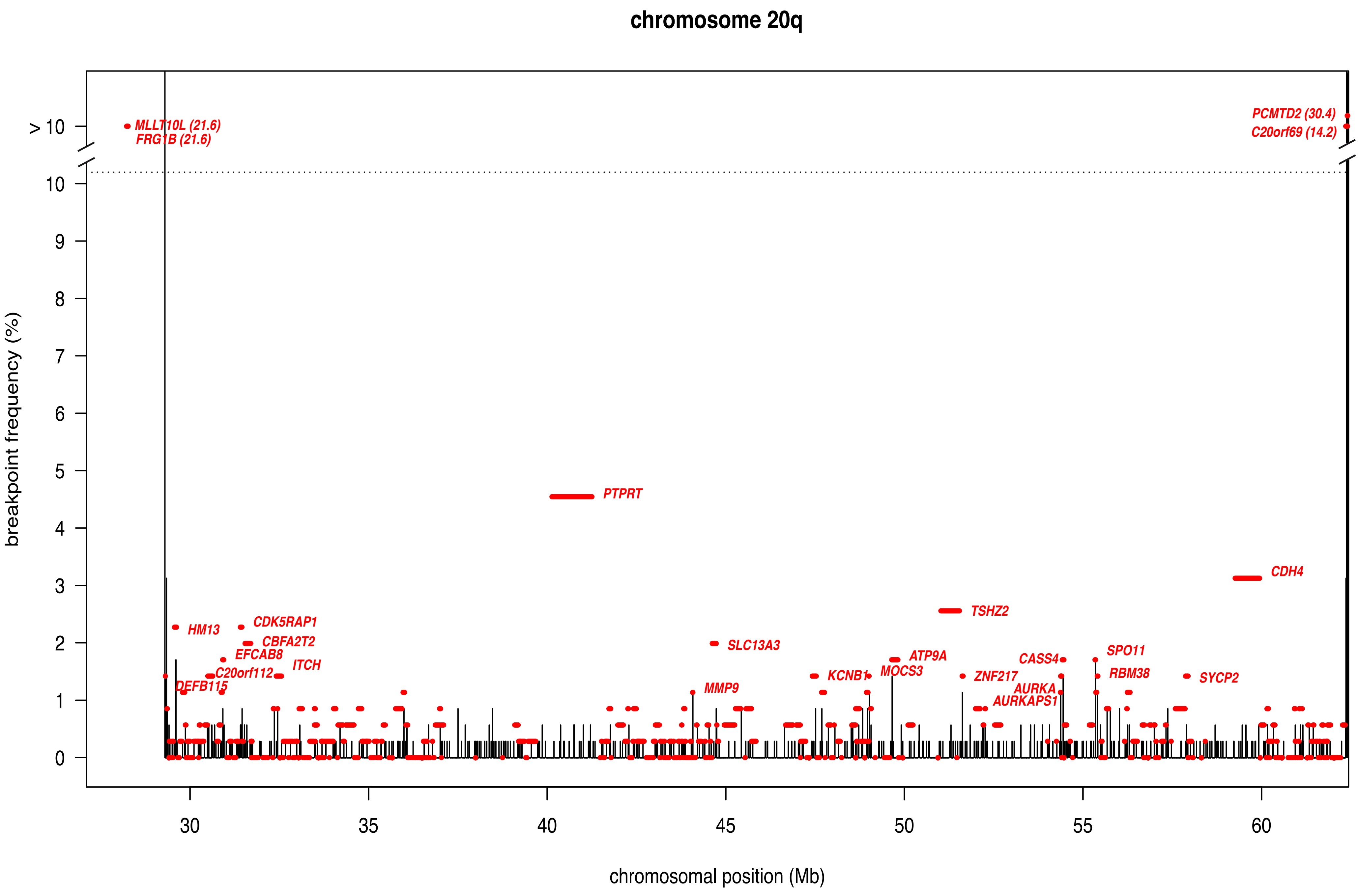

chromosome 20q
breakpoint frequency (%)
chromosomal position (Mb)
> 10
MLLT10L (21.6)
FRG1B (21.6)
PCMTD2 (30.4)
C20orf69 (14.2)
PTPRT
CDH4
TSHZ2
HM13
CDK5RAP1
CBFA2T2
EFCAB8
ITCH
C20orf112
DEFB115
SLC13A3
MMP9
KCNB1
MOCS3
ATP9A
ZNF217
CASS4
AURKA
AURKAPS1
SPO11
RBM38
SYCP2
0
1
2
3
4
5
6
7
8
9
10
30
35
40
45
50
55
60

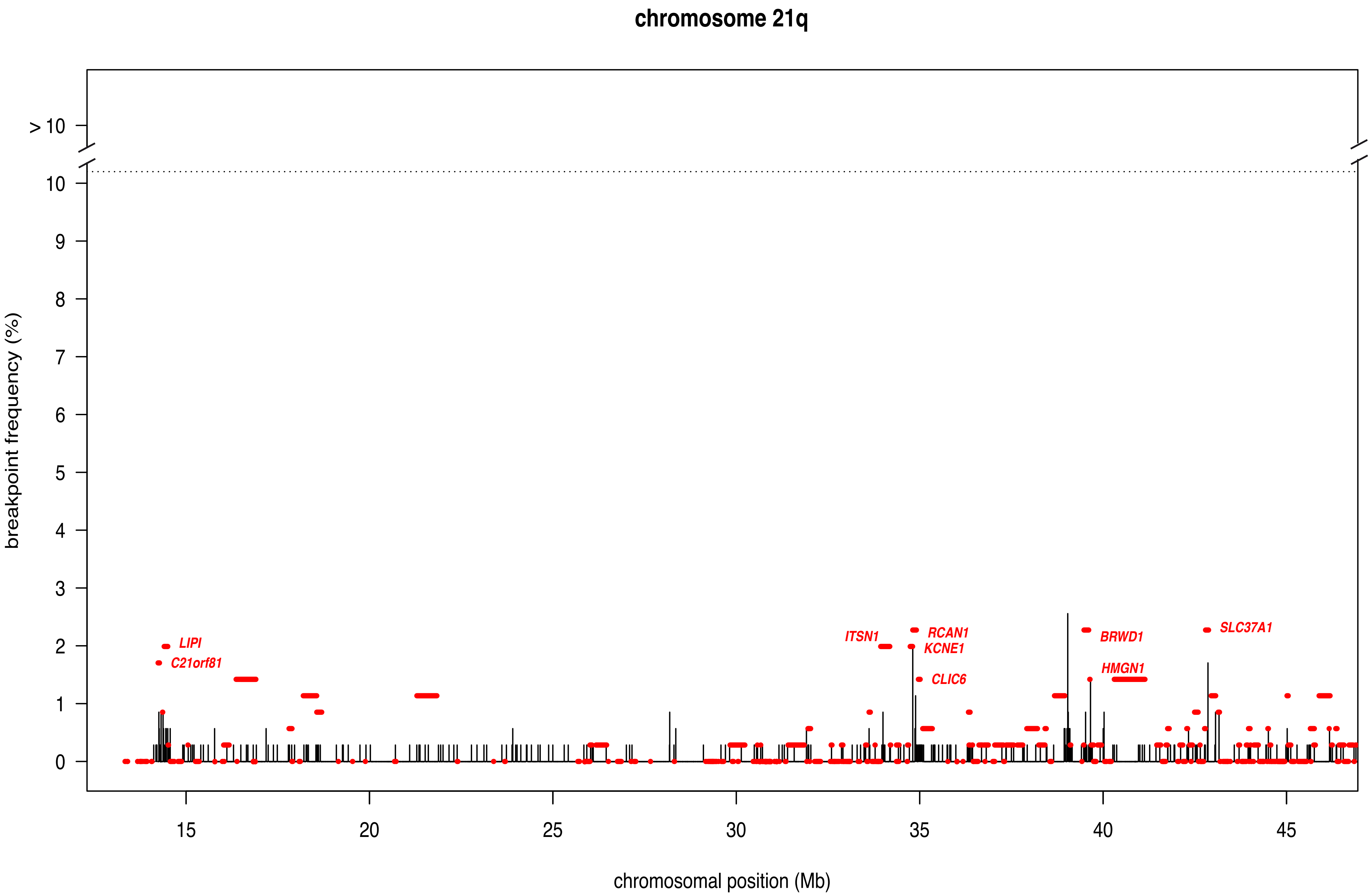
chromosome 21q
> 10
10
9
8
7
6
5
4
3
2
1
0
breakpoint frequency (%)
LIPI
C21orf81
ITSN1
RCAN1
KCNE1
CLIC6
BRWD1
HMGN1
SLC37A1
15
20
25
30
35
40
45
chromosomal position (Mb)

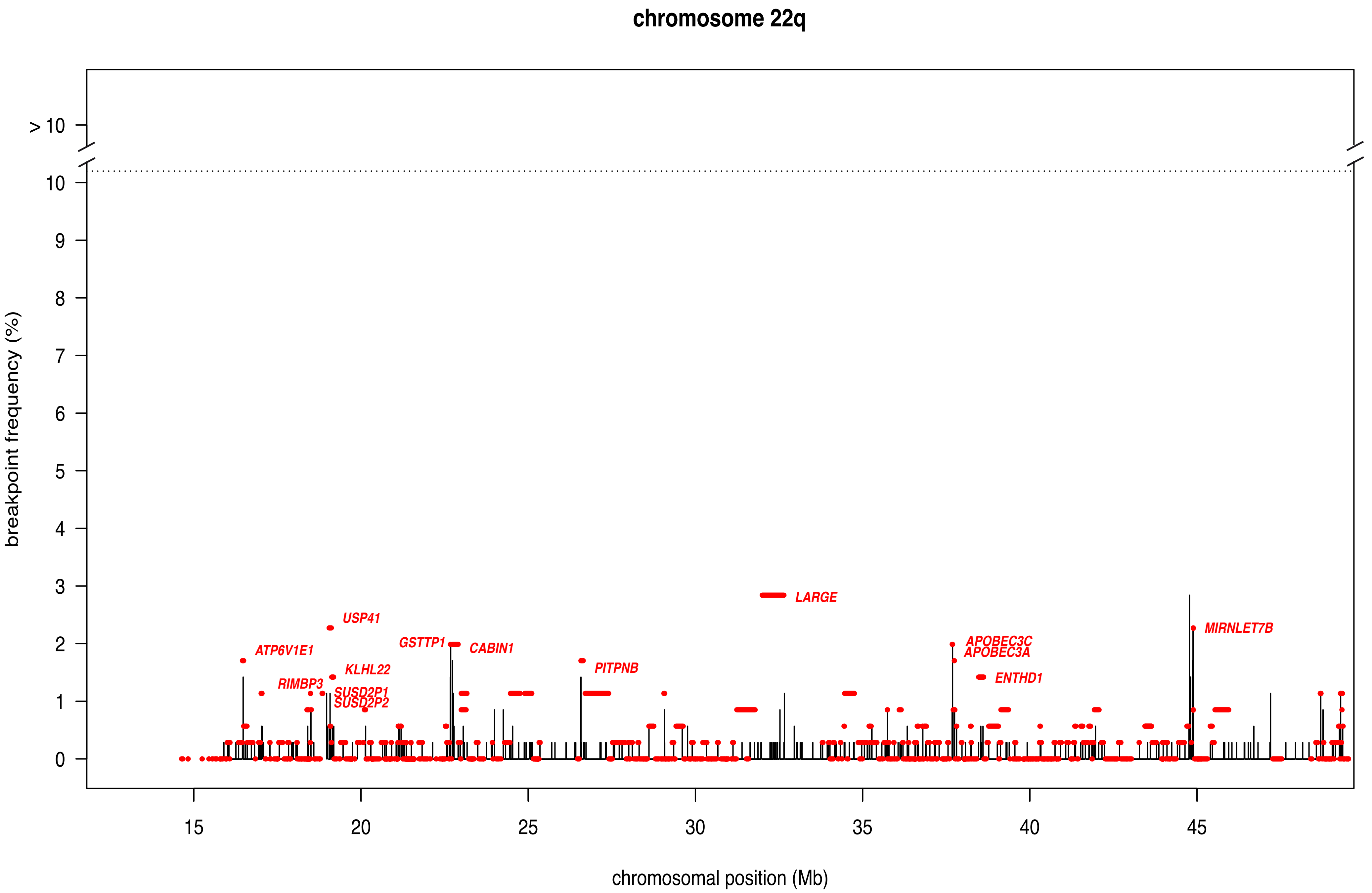
chromosome 22q
breakpoint frequency (%)
chromosomal position (Mb)
> 10
10
9
8
7
6
5
4
3
2
1
0
15
20
25
30
35
40
45
USP41
ATP6V1E1
GSTTP1
CABIN1
KLHL22
RIMBP3
SUSD2P1
SUSD2P2
PITPNB
LARGE
APOBEC3C
APOBEC3A
ENTHD1
MIRNLET7B

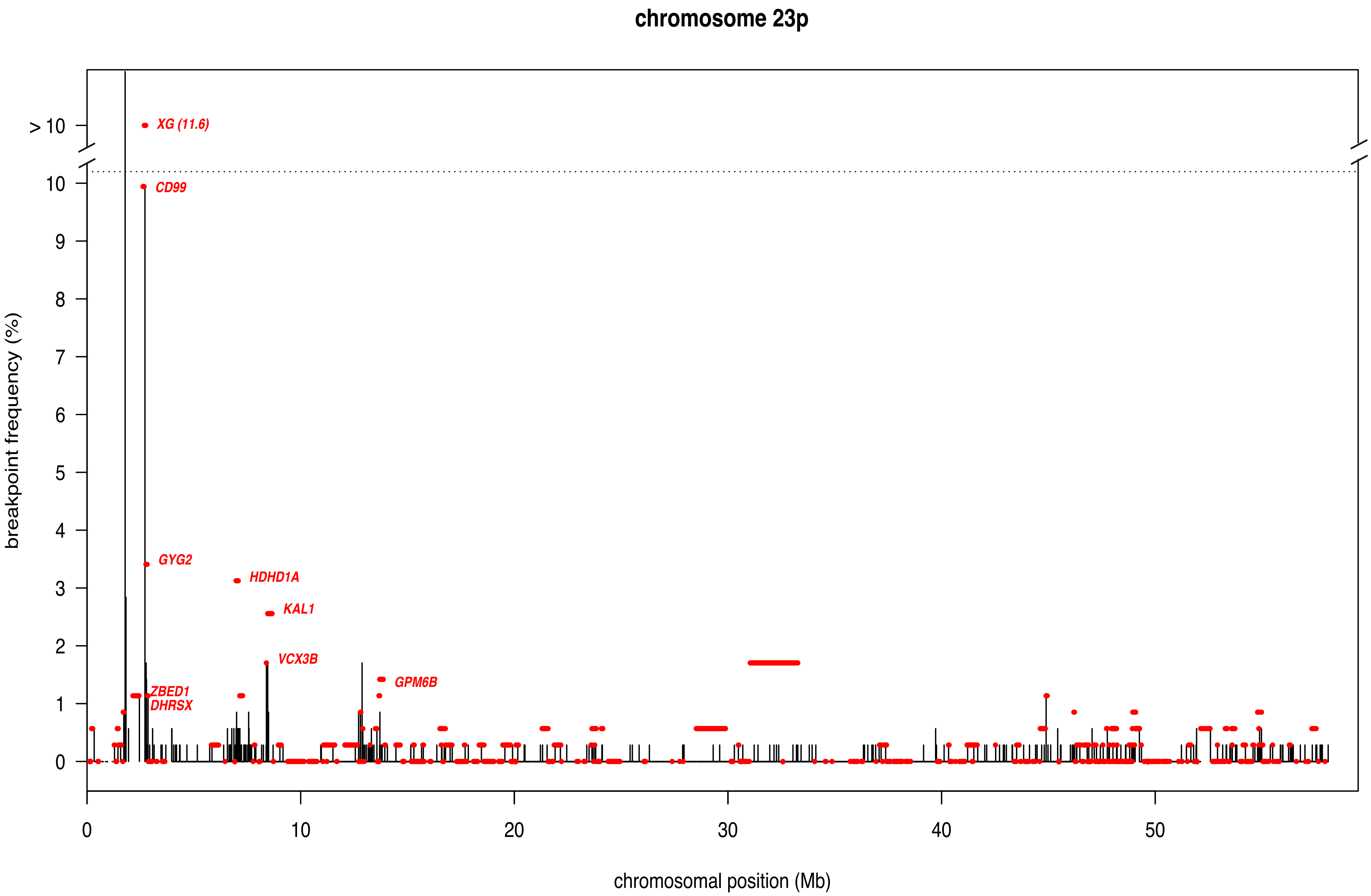
chromosome 23p
breakpoint frequency (%)
chromosomal position (Mb)
XG (11.6)
CD99
GYG2
HDHD1A
KAL1
VCX3B
GPM6B
ZBED1
DHRSX
> 10
10
9
8
7
6
5
4
3
2
1
0
0
10
20
30
40
50

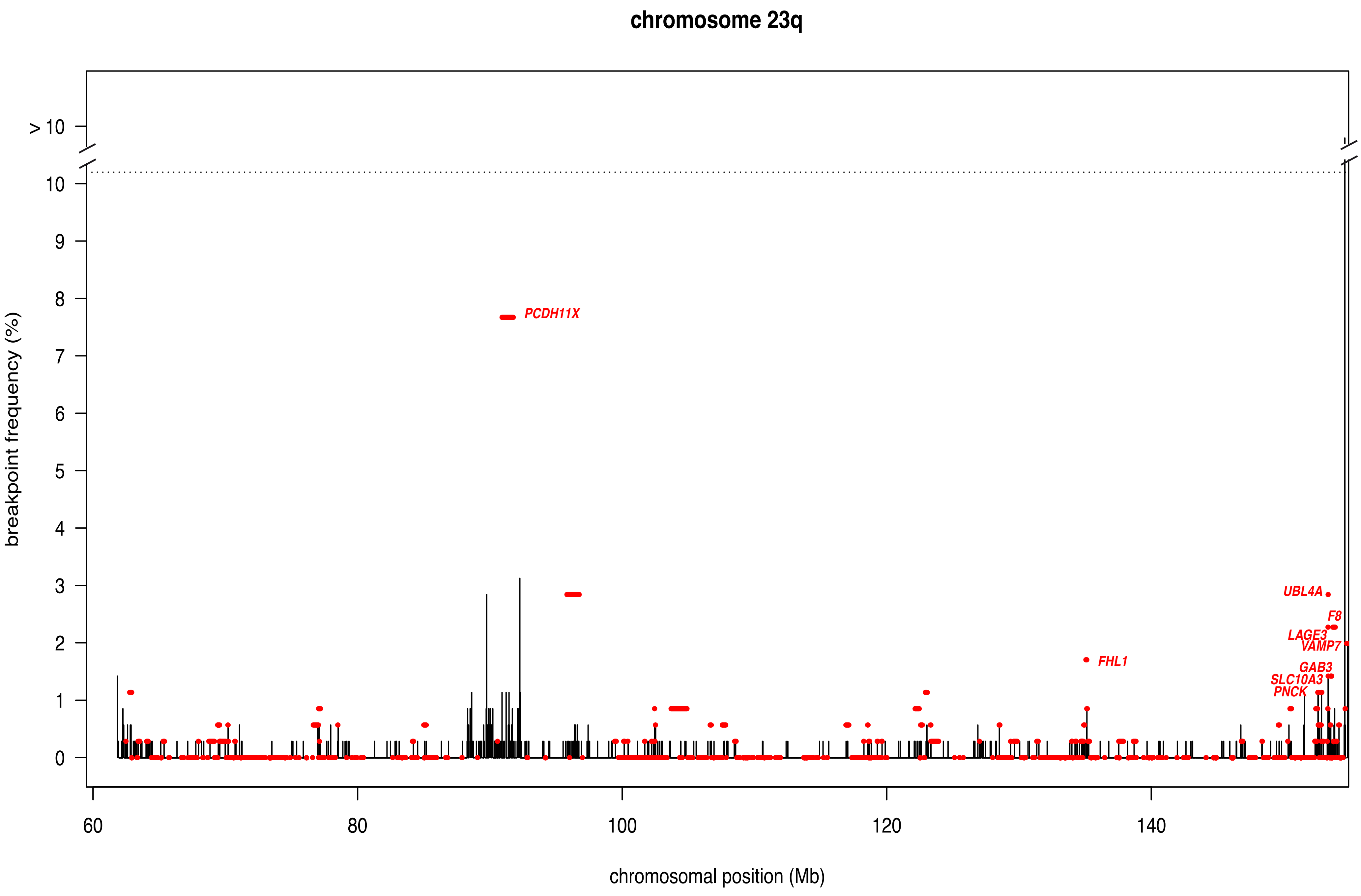
chromosome 23q
breakpoint frequency (%)
chromosomal position (Mb)
> 10
10
9
8
7
6
5
4
3
2
1
0
60
80
100
120
140
PCDH11X
FHL1
UBL4A
F8
LAGE3
VAMP7
GAB3
SLC10A3
PNCK
